# Comprehensive analysis of epigenetic and epitranscriptomic genes’ expression in human NAFLD

**DOI:** 10.1007/s13105-023-00976-y

**Published:** 2023-08-25

**Authors:** Jose M. Herranz, Amaya López-Pascual, Alex Clavería-Cabello, Iker Uriarte, M. Ujúe Latasa, Ainara Irigaray-Miramon, Elena Adán-Villaescusa, Borja Castelló-Uribe, Bruno Sangro, María Arechederra, Carmen Berasain, Matías A. Avila, Maite G Fernández-Barrena

**Affiliations:** 1 Hepatology Laboratory, Solid Tumors Program, CIMA, CCUN, University of Navarra, Pamplona, Spain; 2 CIBERehd, Instituto de Salud Carlos III, Madrid, Spain; 3 Hepatology Unit, CCUN, Navarra University Clinic, Pamplona, Spain; 4 Instituto de Investigaciones Sanitarias de Navarra IdiSNA, Pamplona, Spain

**Keywords:** Non-alcoholic fatty liver disease, Gene expression, Epigenetics, Epitranscriptomics, Metabolism

## Abstract

Non-alcoholic fatty liver disease (NAFLD) is a multifactorial condition with a complex etiology. Its incidence is increasing globally in parallel with the obesity epidemic, and it is now considered the most common liver disease in Western countries. The precise mechanisms underlying the development and progression of NAFLD are complex and still poorly understood. The dysregulation of epigenetic and epitranscriptomic mechanisms is increasingly recognized to play pathogenic roles in multiple conditions, including chronic liver diseases. Here, we have performed a comprehensive analysis of the expression of epigenetic and epitranscriptomic genes in a total of 903 liver tissue samples corresponding to patients with normal liver, obese patients, and patients with non-alcoholic fatty liver (NAFL) and non-alcoholic steatohepatitis (NASH), advancing stages in NAFLD progression. We integrated ten transcriptomic datasets in an unbiased manner, enabling their robust analysis and comparison. We describe the complete landscape of epigenetic and epitranscriptomic genes’ expression along the course of the disease. We identify signatures of genes significantly dysregulated in association with disease progression, particularly with liver fibrosis development. Most of these epigenetic and epitranscriptomic effectors have not been previously described in human NAFLD, and their altered expression may have pathogenic implications. We also performed a comprehensive analysis of the expression of enzymes involved in the metabolism of the substrates and cofactors of epigenetic and epitranscriptomic effectors. This study provides novel information on NAFLD pathogenesis and may also guide the identification of drug targets to treat this condition and its progression towards hepatocellular carcinoma.

**Supplementary Information:**

The online version contains supplementary material available at 10.1007/s13105-023-00976-y.

## Introduction

Non-alcoholic fatty liver disease (NAFLD) is a multifactorial condition with a complex etiology. Its incidence is increasing globally in parallel with the obesity epidemic, and it is now recognized as the most common liver disease in Western countries. The exact prevalence of NAFLD is difficult to determine as it is often asymptomatic, but recent estimates suggest that it affects more than 30% of the general population [117].

**Fig. 1:**
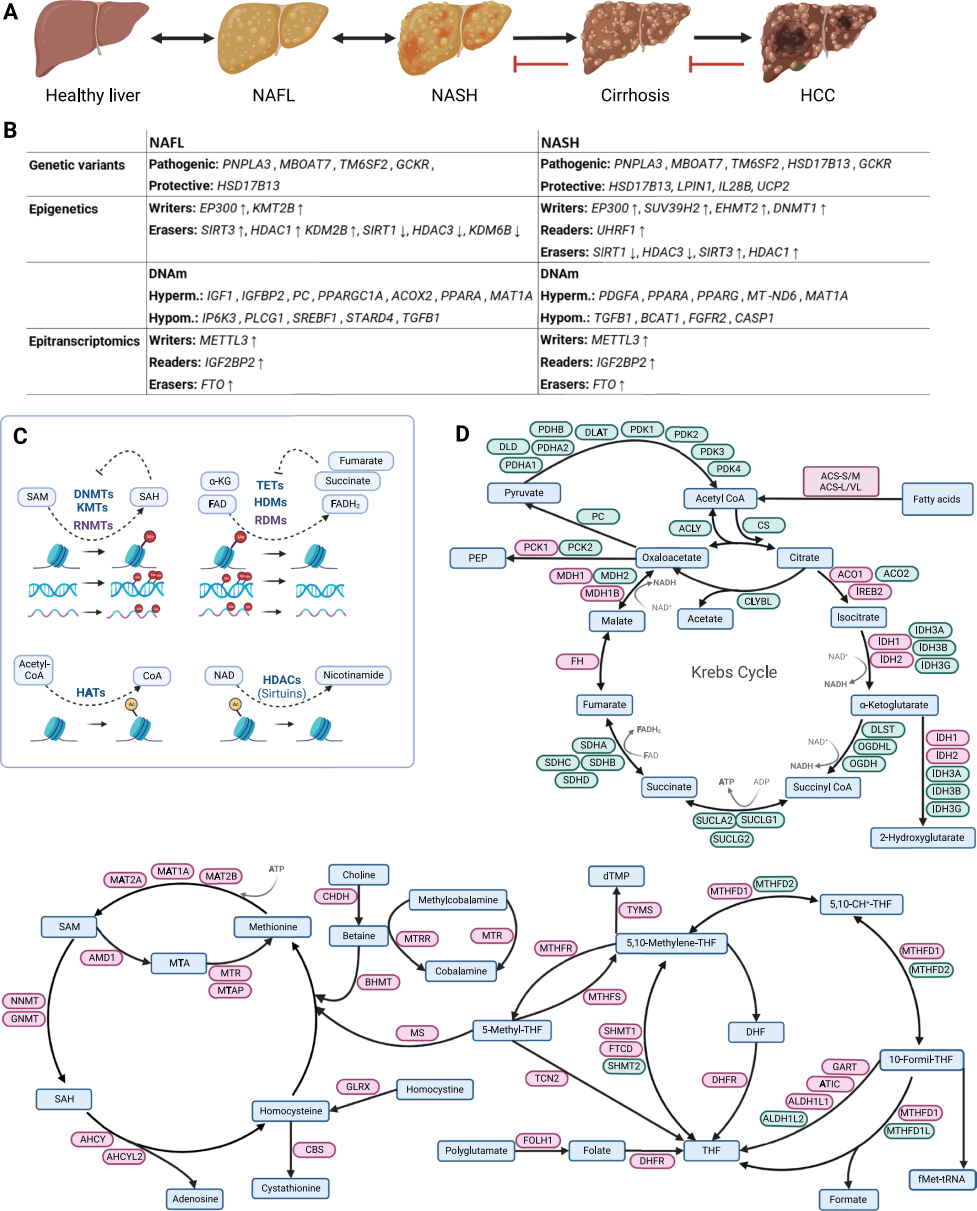
NAFLD progression, genetic variants in NAFLD, and the cross-talk between epigenetic and epitranscriptomic factors and metabolism. **A** Natural history of NAFLD to end-stage liver disease and hepatocellular carcinoma (HCC). **B** Genetic variants with a known pathogenic or protective role for NAFLD, epigenetic and epitranscriptomic genes known to be up- (↑) or downregulated (↓) in NAFLD. The DNA methylation status of genes potentially involved in NAFLD pathogenesis is also indicated. **C** Cross-talk between epigenetic and epitranscriptomic factors and metabolites acting as substrates or inhibitors of these reactions. **D** Major metabolic pathways involved in the synthesis and conversion of substrates and cofactors of epigenetic and epitranscriptomic enzymatic reactions. Abbreviations can be found in Suppl. Table 8

NAFLD encompasses a spectrum of disorders defined by the presence of abnormal lipid accumulation in hepatocytes with or without evidence of liver cell injury [5]. Most patients with NAFLD have non-alcoholic fatty liver (NAFL) defined as simple steatosis or isolated fat accumulation, whereas a subset of NAFL patients can progress to non-alcoholic steatohepatitis (NASH), characterized by steatosis with evidence of hepatocellular injury, inflammation, and fibrosis [5, 10]. The severity of fibrosis correlates with the risk of developing complications like cirrhosis, liver failure, and hepatocellular carcinoma (HCC) [5, 64]. The progression of NAFLD to end-stage liver disease is depicted in Fig. 1A. The precise mechanisms underlying the development and progression of NAFLD are complex and still poorly understood, but a combination of dietary factors, a poor glycemic control, and genetic predisposition likely play a role [27]. Recent evidence suggests that NAFLD is linked with a range of extrahepatic manifestations such as insulin resistance, dyslipidemia, adipose tissue dysfunction, systemic inflammation, and gut microbiota dysbiosis [27]. In addition to these well-known factors related to obesity, type 2 diabetes, and cardiovascular disease, NAFLD is also associated with several other conditions, including chronic kidney disease and metabolic syndrome [7]. Early detection and treatment of NAFLD are essential to prevent its progression towards end-stage liver disease, which has significant implications for both patients and health care systems [116]. NAFL is diagnosed by the presence of steatosis on liver biopsy, whereas the diagnosis of NASH requires the presence of steatosis, necroinflammation, and fibrosis [69]. The most common cause of death in patients with NAFLD is cardiovascular disease, and the risk of death from NAFLD is increased in patients with NASH [69].

Epigenetics refer to the heritable modification of gene expression that does not involve changes in the underlying DNA sequence [33]. These modifications can occur in the form of DNA methylation, histone modification, and non-coding RNA-mediated gene regulation [33]. The field of epigenetics is rapidly evolving and holds great promise for the treatment of various diseases including NAFLD [20, 37, 44, 50]. Emerging evidence suggests that epigenetic alterations may contribute to the development of NAFLD by regulating genes involved in lipid metabolism, inflammation, and cell death [65, 97]. DNA methylation is a common epigenetic modification based on the addition of a methyl group to cytosine residues in CpG dinucleotides [74]. This modification can silence gene expression by preventing the binding of transcription factors to DNA and by promoting chromatin compaction. Several studies have found increased DNA methylation in the liver tissue of patients with NAFLD, particularly in genes involved in lipid metabolism and inflammation [42, 111, 122]. The covalent modification of histones is another epigenetic mechanism that can affect chromatin organization and gene expression. These modifications, which include reactions such as acetylation, methylation, and phosphorylation, can change the structure of chromatin, modulate the recruitment of transcriptional regulators, and influence gene expression. Several studies have found altered histone modification patterns in the liver tissue of patients with NAFLD, which suggests that this epigenetic mechanism may also play a role in the development of this disease [19, 54, 111]. Non-coding RNA-mediated gene silencing is another epigenetic mechanism that can regulate gene expression. MicroRNAs (miRNAs) are a type of non-coding RNA that can bind to complementary sequences in mRNA resulting in gene silencing. Several studies have found altered miRNA expression in the liver tissue of patients with NAFLD [111], suggesting that this epigenetic mechanism may play a role in developing this disease. Overall, the evidence suggests that epigenetic modifications may contribute to the development of NAFLD.

Over the last couple of decades, considerable efforts have been made to characterize the highly dynamic and reversible RNA modifications. More than 150 modifications with regulatory potential have been identified to date and together define the field of epitranscriptomics. Among them, methylation reactions leading to the formation of N6-methyladenosine, N1-methyladenosine, N6,20-O-dimethyladenosine, 5-methylcytosine, and 5-hydroxymethylcytidine are the most extensively investigated [14, 108]. It has become evident that these RNA modifications significantly influence gene expression, and their dysregulation is increasingly being associated with different pathologies including NAFLD [123]. Interestingly, epigenetic and epitranscriptomic mechanisms also rely on reversible enzymatic reactions performed by the so-called epigenetic and epitranscriptomic “writers” and “erasers,” as well as protein-protein and protein-DNA/RNA interactions mediated by “readers,” processes that are amenable to pharmacological targeting [28, 29].

While some gene variants are well-known pathogenic or protective factors in NAFLD (Fig. 1B), the roles of specific epigenetic and epitranscriptomic factors in the progression of this disease remain poorly defined. There are some reports showing the dysregulation in the expression of certain epigenetic and epitranscriptomic factors in NAFL and NASH, as well as altered methylation patterns in key genes for liver disease progression in these conditions (Fig. 1B) [49, 86, 123]. On the other hand, fluctuations in the intracellular levels of specific intermediary metabolites, including those associated with dietary alterations and obesity, can impact on epigenetic and epitranscriptomic mechanisms for which these metabolites behave as substrates or inhibitors [13, 28, 41, 58, 65, 80]. Thus, there is a complex interplay between the intracellular pools of metabolites, related metabolic enzymes, and the epigenetic and epitranscriptomic machinery (Fig. 1C and D). Further studies are needed to confirm the role of epigenetics and epitranscriptomics in NAFLD pathogenesis and to identify potential epigenetic targets for the prevention and treatment of this chronic liver condition. In the present study, we have performed a comprehensive transcriptomic analysis of hepatic epigenetic and epitranscriptomic genes in different cohorts of patients. Our integrated data revealed marked changes in the expression of specific genes in association with the course of the disease. These findings may improve our understanding of NAFLD pathogenesis and pave the way for the identification of novel therapies [93].

## Materials and methods

### Functional selection of interrogated genes

Epigenetic and epitranscriptomic genes were selected from the literature [11] and *Modomics* [12], *EpiFactors* [70], and *ChromoHub* [61] databases to generate a manually curated list. Among the epigenetic genes, those whose function was related to the methylation and acetylation of DNA and histones, as well as the citrullination (or deimination) of histone arginine residues, were considered conventional epigenetic genes. All other epigenetic genes were classified as non-conventional epigenetic genes. Then, the function of each selected gene was confirmed by the availability in reliable databases (GeneCards, PubMed, and Uniprot) of experimental evidence demonstrating their purported biochemical activity. All the genes with no experimental evidence of functional activity were discarded. Reader and eraser functions were prioritized above reader or cofactor activity to classify the genes, although some of them have several functions as readers and erasers or writers [11]. On the other hand, genes encoding for metabolic enzymes involved in the metabolism of epigenetic cofactors were selected based on the proximity of function to the epigenetic and epitranscriptomic machinery.

### Transcriptomic data preprocessing

Transcriptomic high-throughput data (RNA-seq) were downloaded from the NCBI Sequence Read Archive (SRA) in fastq format using *SRA Toolkit* version 3.0.0. The first step in the workflow involves quality control and preprocessing of the raw RNA-seq data. Adapter sequences and low-quality reads are removed using *TrimGalore* version 0.6.0 with *Cutadapt* version 1.18 [53, 71]. Subsequently, clean reads are aligned to the reference genome using a splice-aware aligner *STAR* version 020201 over genome version hg38 [26]. Aligned reads are then quantified at the gene level using *HTseq* version 0.11.0 [4]. *EdgeR* version 3.28.1 [90] for R software version 3.6.3 (hereafter referred to as R) requires raw read counts as input and performs normalization using the trimmed mean of *M*-values (TMM) method [91]. TMM normalization accounts for library size differences and composition biases, ensuring accurate comparisons between samples.

A “normal liver” was considered when a sample is collected from a healthy liver (without NAFLD, alcoholic hepatitis, virus infection, or cancer). However, the original sequencing studies may have not reported other non-hepatic diseases that could affect the liver.

### Data integration

Once all samples were correctly normalized, *ComBat* [47] from *sva* R package version 3.44.0 [56] was used to remove the batch effect using sex and disease stage as covariates, the only variables present in all samples or inferable (sex). *ComBat* integrates and harmonizes high-dimensional biological data from multiple sources by removing batch effects, such as differences in experimental protocols while preserving true biological differences. The method consists on organizing data into a single matrix, identifying batch effects, fitting a linear model that includes both biological factors and batch effects, and using an empirical Bayes framework to estimate model parameters. *ComBat* then computes adjusted values for each feature, subtracting the estimated batch effect from the original data. The resulting dataset, with reduced batch effects, can be used for downstream analyses more accurately and reliably.

In addition, the adjusted expression matrix was transformed into a positive value matrix adding the minimum value of expression of genes that were negative in some of the samples. Some of the genes were not detected in any of the RNA-seq datasets of liver samples although they could be detected by microarray technology as far as there is no competence between the mRNA molecules. These genes were discarded for not being present in none of the samples of a complete dataset, which inclusion may result in a loss of statistical power and robustness.

### Sample clustering and visualization

The robustness of the batch effect removal was tested using four different dimensional reduction techniques including principal component analysis (PCA), uniform manifold approximation and projection (UMAP), and *t*-distributed stochastic neighbor embedding analysis (*t*-SNE) with M3C version 1.18.0 [46] and discriminant analysis of principal components (DAPC) (*adegenet* version 2.1.7) [48]. UMAP excels in preserving the topological structure of the data by approximating the manifold on which it resides and projecting it onto a lower-dimensional space. Its primary advantage lies in its ability to maintain both local and global structures, making it particularly useful for analyzing complex data. On the other hand, *t*-SNE focuses on minimizing the divergence between two probability distributions: one representing pairwise similarities in high-dimensional space and the other in low-dimensional space. By employing a *t*-distribution to model similarities in the latter, *t*-SNE effectively addresses the crowding problem often encountered in other techniques [105]. Its strength lies in revealing intricate, nonlinear structures in the data and generating visually interpretable embeddings. Meanwhile, PCA is a linear dimensionality reduction technique that identifies the principal components or axes in high-dimensional space where data variance is maximized. By projecting data onto the first few principal components, PCA effectively reduces dimensionality while retaining as much original variance as possible. This technique is particularly suited for datasets with linear relationships and Gaussian-like distributions.

Finally, to complete these unsupervised approximations (clustering with unlabeled data), supervised DAPC was carried out. The number of principal components (PC) selected explained 95% of the variance. All the linear discriminants generated were taken because the number of eigenvalues was small enough to make the difference at this point. The explained intergroup variance of each gene of the signature was taken as a variable importance measure, to uncover the most informative genes (genes that explain more than 2% of the variance). The supervised mode of DAPC was also used to reconstruct the evolution of the disease with the whole transcriptome following the same parameter choice, checking if it was congruent with the development described in the bibliography. Using gene set variation analysis (GSVA) package version 1.44.2 [35] with the implemented single-sample gene set enrichment analysis (ssGSEA) method [55], the pathway activity score of a combination of two previously published gene signatures of NAFLD was computed [36, 92]. This activity score was comprised by the combined list of genes divided into two signatures: the first one, with the genes described to be upregulated in the published signatures, and the second one with those described to be downregulated with disease progression. Thereby, this analysis proved the biological relevance of integrated data.

### Gene expression analyses

To evaluate transcriptomic differences of these epigenetic, epitranscriptomic, and metabolic genes, *edgeR* fits a generalized linear model (GLM) to the normalized count data, using the negative binomial distribution to account for both biological and technical variability. Low expression genes were filtered using the *filterByExpr* function implemented in *edgeR* with the default settings*.* Dispersion parameters are estimated using the empirical Bayes method, and statistical tests are performed using the likelihood ratio test or the quasi-likelihood *F*-test. Multiple testing correction is applied using the Benjamini-Hochberg procedure to control the false discovery rate (FDR).

Regarding the number of differentially expressed genes described throughout the present study, only statistically significant changes were reported for each comparison. To clarify the possible molecular and biological functions of differentially expressed genes, gene set enrichment analysis (GSEA) was conducted using *clusterProfiler* R package version 4.4.4 [118] with whole *msigdb* database [99]. Between 200 and 800 significant genes were taken, ordered by the log2 fold change, as input for pathway analyses. The influence of age, sex, and fibrotic stage was tested, and the presence of a potential confounding effect was ruled out.

### Hierarchical clustering

Hierarchical clustering of conventional epigenetic genes using Euclidean distance in combination with Ward’s clustering method, with *dist* function in R over adjusted read counts, resulted in three gene sets or clusters. The most informative and changing cluster was selected for the signature definition from epigenetic and epitranscriptomic gene lists using GSVA. Gene signatures revealed a gradient of expression in both disease stages that, for simplicity of downstream comparisons, were subclassified using the same hierarchical clustering method in three subgroups (low, medium, and high expression levels). This approach resulted in three groups per signature in each disease stage (NAFL and NASH).

### Statistical analyses

Genes were considered differentially expressed if the adjusted *p*-value (FDR method of Benjamini and Hochberg) was lower than 0.05. For exploratory pathway analyses, all genes with *p* < 0.05 were included. The identified categories were considered significant and up- or downregulated if *p*-value < 0.05. Gene expression correlation was computed using Pearson’s correlation methods, and significantly correlating gene pairs were plotted using *corrplot* package version 0.92.

For the statistical significance of more than two groups, the Kruskal-Wallis test was applied. Multiple comparisons were controlled by the false discovery rate (FDR) using the Benjamini and Hochberg correction (*Q* = 5%). Chi-square tests of homogeneity were used to analyze if the observed proportion of patients in a certain fibrosis stage was distributed as in the integrative dataset (used as expected proportions). Gene signature association with HCC patients’ survival was measured with the survival package in R v3.3-5 as described [104]. HCC gene expression and patients’ related information were obtained from data generated by the TCGA research network (https://www.cancer.gov/tcga). GraphPad Prism 9.0.2 software (GraphPad Prism, San Diego, CA, USA) was used for these statistical analyses and the corresponding boxplots. Data are presented as mean and standard error. Values of *p* < 0.05 were considered statistically significant.

## Results

### Integration of transcriptomic data

**Fig. 2:**
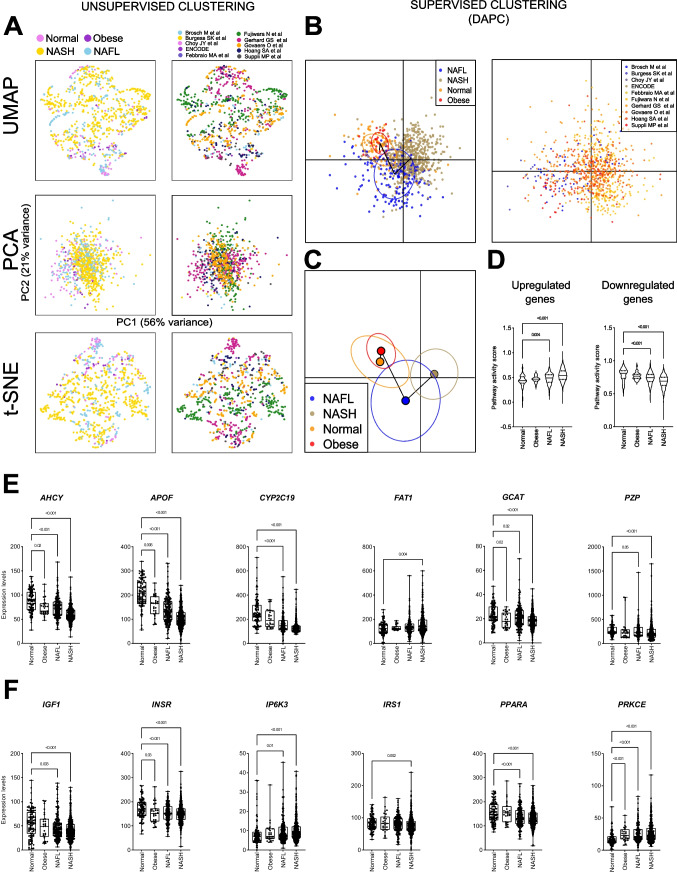
Bioinformatic and biological validation of the integrated transcriptomic data from human liver gene expression datasets. **A** Dimensionality reduction analyses using *t*-SNE, UMAP, and PCA unsupervised techniques. **B** Supervised dimensionality reduction by DAPC represented as a scatterplot using disease stage as groups (left) and the source (published article, right). **C** Supervised DAPC representing the centroid and ellipses of 95% confidence interval joined by the maximum similarity path representing the natural history of the disease. **D** Pathway activity score performed using previously published NAFLD signatures [36, 92]. **E** Expression of genes previously reported in NAFLD gene signatures in our integrated transcriptomic datasets. **F** Expression of genes functionally linked to NAFLD pathogenesis in our integrated datasets. Transcriptomic data are expressed as the trimmed mean of *M*-values (TMM) and grouped according to liver disease classification (Normal, Obese, NAFL, and NASH). *p*-values were obtained from the Kruskal-Wallis test and adjusted by FDR Benjamini and Hochberg correction (**E** and **F**). Values of *p* < 0.05 were considered statistically significant

The transcriptomic data of 903 human liver samples were integrated from 10 publicly available datasets (Table 1), including samples from normal (*n* = 103, 11%), obese (*n* = 27, 3%), NAFL (*n* = 194, 21%), and NASH (*n* = 579, 64%) patients. The dimensionality reduction analyses using *t*-SNE, UMAP, and PCA techniques revealed a strong tendency for liver samples to cluster by disease stage (Fig. 2A). This finding suggests that the batch effect was successfully mitigated, enabling a more accurate representation of the underlying biological processes. In addition to the unsupervised dimensionality reduction techniques, a supervised DAPC conducted to test if samples would be grouped by independent datasets demonstrated that the batch effect was diminished even using the supervised mode over the integrated data (Fig. 2B). Supervised DAPC based on the similarity of the full transcriptional profile was able to reconstruct the natural history of NAFLD (Fig. 2C). This approach recapitulates at the transcriptional level what is described in the literature in the transition from normal liver, and livers from obese patients, towards NAFL and finally NASH stages. We also found that the activity scores using previously published NAFLD signatures [36, 92] in our integrated data clearly reflected the progression of the disease (Fig. 2D). The soundness of our approach was further supported by the analysis of specific genes within previously reported NAFLD gene signatures [36, 92] (Fig. 2E and Suppl. Fig. 1A), as well as genes functionally linked to NAFLD pathogenesis (Fig. 2F) [1, 2, 31, 36, 78, 92]. As mentioned above, liver fibrosis is a hallmark of NASH progression [10, 109], and consistently, we observed that the expression of a broad range of these genes significantly changed according to the fibrosis stage of liver tissues (F0 to F4) [10] in the NASH cohort (Suppl. Fig. 1B). Altogether, these analyses confirm the robustness of our strategy and support the accuracy of further gene expression studies performed in this integrated dataset.

**Table 1:** Studies included for the meta-analysis of human liver transcriptomic data, integrated from publicly available datasets. Sequence Read Archive (SRA) https://www.ncbi.nlm.nih.gov/sra

First author	SRA ID	Normal	Obese	NAFL	NASH	Total
Brosch M et al.	SRP120376	12	15	15	15	57
Burgess SK et al.	SRP135703	20	0	0	0	20
Choy JY et al.	SRP058036	2	0	0	0	2
ENCODE	SRP003497	2	0	0	0	2
Febbraio MA et al.	SRP149518	3	0	3	3	9
Fujiwara N et al.	SRP353346	0	0	12	258	270
Gerhard GS et al.	SRP174668	36	0	50	106	192
Govaere O et al.	SRP217231	10	0	51	155	216
Hoang SA et al.	SRP197353	4	0	48	26	78
Suppli MP et al.	SRP186450	14	12	15	16	57
Total (*n* of patients)		**103**	**27**	**194**	**579**	**903**

### Differential gene expression of epigenetic and epitranscriptomic genes

Epigenetic factors comprising 20 families, a total of 419 genes, were selected for the analyses. Of these, 257 genes (11 families) were categorized as conventional epigenetic factors (Table 2). These are considered the most widely described genes that belong to three different categories of epigenetic writers: DNA methyltransferases (DNMTs), protein arginine-methyltransferases (PRMTs), protein lysine-methyltransferases (KMTs), histone acetyl-transferases (HATs); epigenetic erasers: DNA demethylases (TETs), histone-lysine demethylases (HDMs), histone deacetylases (HDACs), and histone deiminases (HDIs); and epigenetic readers: DNA methyl-binding proteins (MBPs), histone methyl readers (HMRs), and histone acetyl readers (HARs). Other 162 genes (9 families) were categorized as non-conventional epigenetic factors (Table 2). These included chromatin remodeling factors (ChrRs) and additional writers, erasers, and readers with other epigenetic activities such as histone-tyrosine phosphatases (HTPs), histone deubiquitylases (HDUs), or phosphorylated histone readers (PHRs) (Table 2). Regarding the epitranscriptomic factors, 6 families involving 137 genes were selected for further analyses, equally comprising the three different categories of epitranscriptomic writers: RNA methyltransferases (RNMTs), pseudo-uridine synthases (PUSs); epitranscriptomic erasers: RNA demethylases (RDMs), RNA hydroxylases (RNHLs); epitranscriptomic readers: methylated RNA readers (MRRs) and others with miscellaneous epitranscriptomic functions, such as RNA acetylation or RNA decapping (Table 2). The classification, function, target, and source of information for selected epigenetic and epitranscriptomic factors are described in Suppl. Table 1 and Suppl. Table 2, respectively. From 556 genes successfully validated as epigenetic and/or epitranscriptomic effectors, 506 were present in all 10 datasets interrogated. Genes not detected in the expression analysis were independently checked set by set without integration. None of them was present in most of the datasets, thereby concluding that their expression in the liver was residual or not present. Out of 419 selected epigenetic genes, 379 were detected, whereas 128 out of 137 epitranscriptomic genes were expressed in the selected transcriptomic datasets.

**Table 2:** Epigenetic and epitranscriptomic genes classified by families. Epigenetic genes are categorized as conventional or non-conventional

Group	Family	Full name	Genes
Conventional epigenetic genes	DNMTs	DNA methyltransferases	3
Conventional epigenetic genes	TETs	Tet methylcytosine dioxygenases	3
Conventional epigenetic genes	MBPs	Methylated CpG binding proteins	18
Conventional epigenetic genes	PRMTs	Arginine (R) methyltransferases	9
Conventional epigenetic genes	KMTs	Lysisne (K) methyltransferases	42
Conventional epigenetic genes	HDMs	Histone demethylases	29
Conventional epigenetic genes	HMRs	Histone methyl readers	77
Conventional epigenetic genes	HATs	Histone acetyl transferases	23
Conventional epigenetic genes	HDACs	Histone deacetylases	17
Conventional epigenetic genes	HARs	Histone acetyl readers	33
Conventional epigenetic genes	HDIs	Histone deiminases	3
Non-conventional epigenetic genes	STKs	Serine threonine kinases	21
Non-conventional epigenetic genes	HTPs	Histone tyrosine phosphatases	4
Non-conventional epigenetic genes	PHRs	Phosphorylated histone readers	3
Non-conventional epigenetic genes	HUbs	Histone ubiquitin ligases	28
Non-conventional epigenetic genes	HDUs	Histone deubiquitylases	5
Non-conventional epigenetic genes	HUbRs	Histone ubiquitin readers	4
Non-conventional epigenetic genes	HMCs	Histone modification cofactors	75
Non-conventional epigenetic genes	ChrRs	Chromatin remodelling factors	6
Non-conventional epigenetic genes	Others	Miscellaneous	17
Epitranscriptomic genes	RNMTs	RNA methyltransferases	65
Epitranscriptomic genes	RDMs	RNA demethylases	5
Epitranscriptomic genes	MRR	Methylated RNA readers	19
Epitranscriptomic genes	PUSs	Pseudouridine synthases	10
Epitranscriptomic genes	RNHLs	RNA hydroxylases	4
Epitranscriptomic genes	Others	Miscellaneous	34

**Fig. 3:**
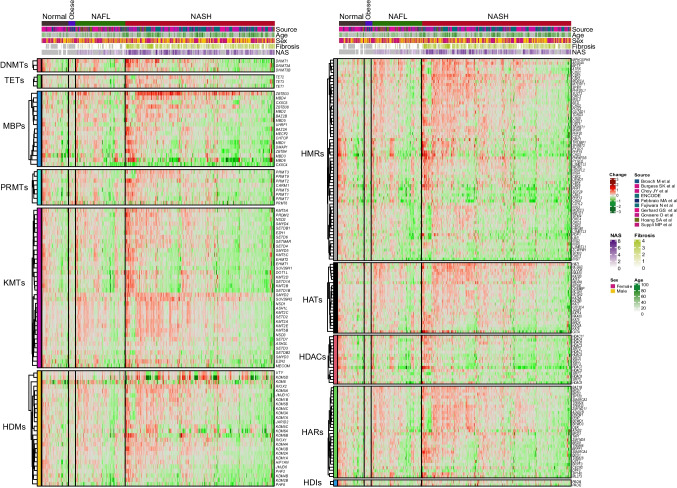
Heatmap of the expression of conventional epigenetic genes grouped in families and according to liver disease classification (normal liver, liver from obese patients, NAFL, and NASH). Expression fold change is compared with normal liver

As can be observed in Fig. 3 and Suppl. Fig. 2, among the 379 epigenetic genes examined (213 conventional, and 133 non-conventional, respectively), profound alterations in their expression were observed for many of them across the different disease stages. When comparing NAFL liver with normal liver, 39 genes (28 conventional and 11 non-conventional) were significantly changed, 12 upregulated (10 conventional and 2 non-conventional) and 27 downregulated (18 conventional and 9 non-conventional). Between NASH and normal liver, we found an increase in the number of differentially expressed genes, 108 of them (62 conventional and 46 non-conventional) were significantly altered, 67 upregulated (35 conventional and 35 non-conventional) and 41 downregulated (27 conventional and 14 non-conventional). The expression of some of these epigenetic effectors, such as *DNMT1*, *SIRT1*, *SIRT3*, *PHF2*, and *ZBTB33*, has been previously described to change in NAFL and NASH, confirming the robustness of our findings [18, 32, 79, 86]. We also identified other genes not previously reported to be dysregulated in NAFLD, such as *HAT1*, *SMYD2*, *CBX5*, *CBX1*, and *MPHOSPH8* which were induced, and *KAT8* which was downregulated (Suppl. Fig. 3A).

**Fig. 4:**
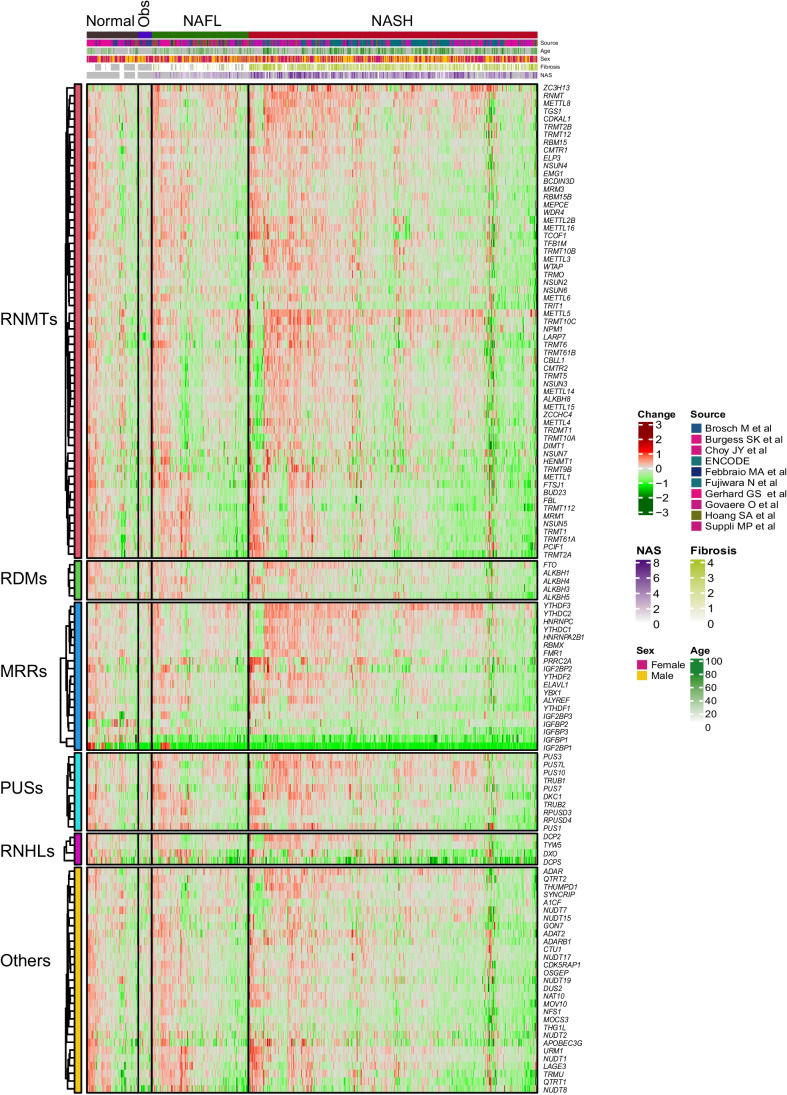
Heatmap of the expression of epitranscriptomic genes grouped in families and according to liver disease classification (normal liver, liver from obese patients, NAFL, and NASH). Expression fold change is compared with normal liver

Focusing on epitranscriptomic genes, 128 genes were interrogated of which 13 were differentially expressed (2 upregulated and 11 downregulated) in NAFL, while in NASH 40 genes were differentially expressed (12 upregulated and 28 downregulated) when compared to normal liver samples (Fig. 4). Among these genes, *YTHDF3*, *YTHDC2*, *RNMT*, *METTL5*, *IGFBP3*, and *TRMT10C* were upregulated in both NAFL and NASH liver tissues, while *IGFBP1* expression was downregulated, in agreement with previous reports [34] (Suppl. Fig. 3B).

Despite these changes in both types of genes, the total number of them that showed statistically significant up- or downregulation in the NAFL or NASH cohorts was proportionally low. Interestingly, upon closer examination, for both epigenetic and epitranscriptomic genes, there seemed to be subgroups of patients within the NAFL and NASH cohorts that presented similar gene expression patterns (Figs. 3 and 4).

### Epigenetic and epitranscriptomic gene signatures

**Fig. 5:**
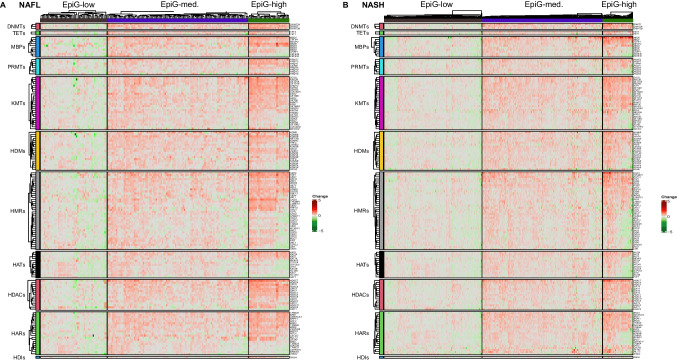
Heatmap showing the classification of patients according to their epigenetic gene expression signature. **A** NAFL and **B** NASH patients. Samples were grouped using hierarchical clustering based on similar expression profiles. Clusters of gene expression are classified as of low (EpiG-low), medium (EpiG-med), and high (EpiG-high) expression. Fold change is established comparing with EpiG-low

**Fig. 6:**
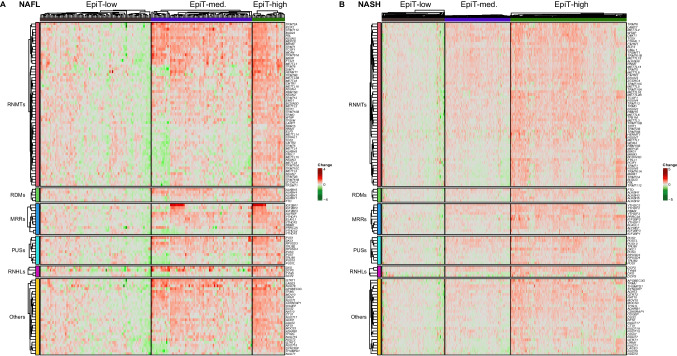
Heatmap showing the classification of patients according to their epitranscriptomic gene expression signature. **A** NAFL and **B** NASH patients. Samples were grouped using hierarchical clustering based on similar expression profiles. Clusters of gene expression are classified as of low (EpiT-low), medium (EpiT-med), and high (EpiT-high) expression. Fold change is established comparing with EpiT-low

In view of this apparent existence of different subgroups of patients, we next aimed to define the gene signatures that could stratify them. To this end, the observed expression gradients were split using hierarchical clustering on Euclidean distance and three subgroups of patients with low (EpiG-low), medium (EpiG-med), and high (EpiG-high) expression in each signature were generated. Taking gene expression as input for the hierarchical clustering, the Euclidean distance was used as a similarity measure to define specific signatures that captured the differences observed among patients with NAFL or NASH. This signature comprised 188 and 193 genes for NAFL and NASH patients, respectively. To increase the robustness of the stratification, only genes expressed in both disease stages and upregulated in the third cluster (EpiG-high) were selected in the final epigenetic signature, which included a total of 156 conventional epigenetic genes (Fig. 5A and B, and Suppl. Table 3). To define the epitranscriptomic signature the same process was carried out, and three subgroups were defined: EpiT-low, EpiT-med, EpiT-high. In this case, the signature included 123 and 119 genes for NAFL and NASH patients, respectively. When only genes expressed in both disease stages, and upregulated in the third cluster (EpiT-high), were considered, a more robust signature with 119 genes was established (Fig. 6A and B, and Suppl. Table 3). The distribution of male and female NASH patients in the EpiG and EpiT-low, EpiT-med, and EpiT-high clusters was homogeneous (Supplementary Fig. 4).

**Fig. 7:**
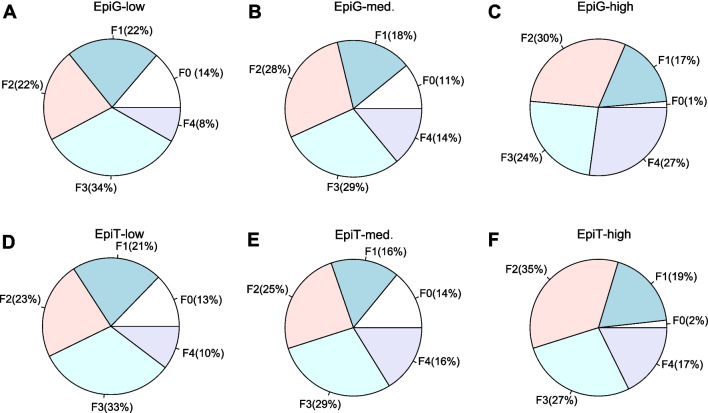
Pie charts showing the proportion of patients within each fibrosis stage (F0–F4) in **A** EpiG-low, **B** EpiG-medium, **C** EpiG-high, **D** EpiT-low, **E** EpiT-medium, and **F** EpiT-high clusters

Next, we evaluated if the differential expression of epigenetic and epitranscriptomic genes could be related to the pathological characteristics of the tissues. Therefore, we tested the distribution of patients according to their fibrosis stage (F0–F4) in the different Epi-G and Epi-T subgroups. The proportion of patients in each fibrosis stage was calculated in the full integrative dataset according to the information on biopsy-proven fibrosis as reported by the authors. Thus, 11% of samples from NASH patients showed no fibrosis (F0), 19% of patients corresponded to F1 fibrosis stage, 26% had F2 fibrosis, 30% had F3 fibrosis, and 13% of patients were at F4 fibrosis stage. Then, we examined the distribution of F0–F4 cases across the groups of NASH patients classified in the different epigenetic (EpiG) and epitranscriptomic (EpiT) subgroups. Our analysis revealed an unbalanced distribution of patients classified by fibrosis stage across the EpiG groups, with patients classified as EpiG-high including significantly less F0 cases and the highest proportion of patients with advanced fibrosis (F4) (Fig. 7A–C). Although less marked, a similar observation was made when patients were classified according to EpiT category. F0 cases were very few, while F4 patients were more frequent in the EpiT-high subgroup compared to EpiT-low (Fig. 7D, E).

**Fig. 8:**
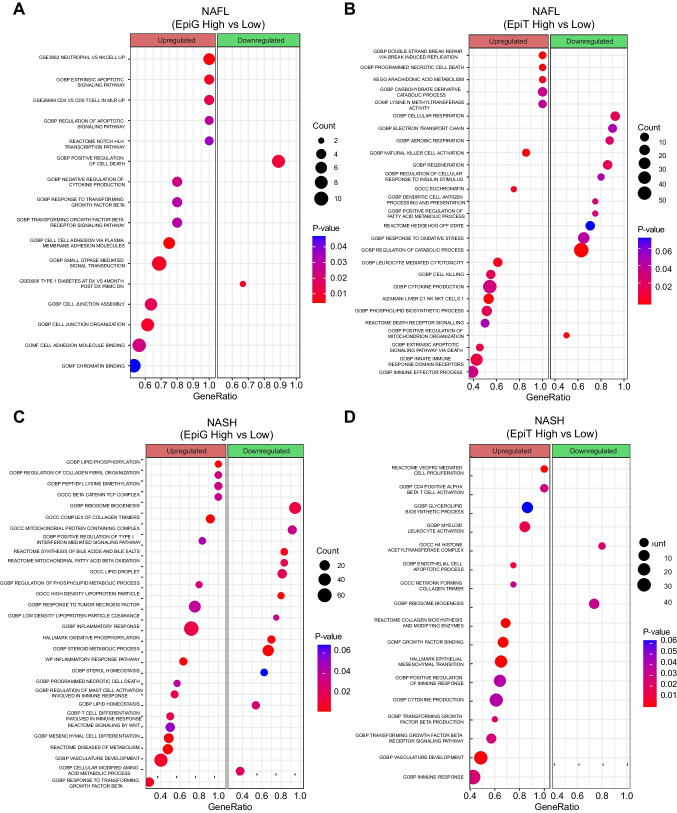
Pathway analyses performed on the differentially expressed genes in the epigenetic and epitranscriptomic “high” and “low” signatures within NAFL and NASH liver samples. **A** Pathway analysis performed on differentially expressed genes between EpiG-low and EpiG-high clusters in NAFL patients, and **B** in NASH patients. **C** Pathway analysis performed on differentially expressed genes between EpiT-low and EpiT-high clusters in the NAFL patients, and **D** in NASH patients

To explore the molecular landscape and biological characteristics of the NAFL and NASH liver tissue samples classified according to their EpiG and EpiT profiles, we performed Gene Ontology (GO) and gene set enrichment analysis (GSEAs) on the genes differentially expressed (DEGs) between these subgroups (Fig. 8A and B). In the NAFL stage, while the analysis of gene expression among the epigenetic subgroups uncovered relevant processes such as apoptotic signaling, events related to cell adhesion, cell death, or processes involving Notch, TGFβ, and other signaling pathways mediated by GTPases (Fig. 8A and Suppl. Table 4), it was the epitranscriptomic subgroups that exhibited a greater number of significant differences. EpiT-high vs EpiT-low subgroups differences encompassed crucial biological functions associated with the pathophysiology of the disease and its progression. Specifically, individuals classified as EpiT-high displayed enrichments in metabolic pathways (carbohydrates and lipids metabolism, cellular respiration), DNA damage and apoptosis-related mechanisms, and, remarkably, numerous inflammatory events (Fig. 8B and Suppl. Table 5). When considering the NASH stage, the comparison between epigenetic subgroups (EpiG-high vs EpiG-low) revealed greater number of differences than in the NAFL cohort. Notably, differentially expressed genes were primarily associated with important metabolic events (lipids, lipoproteins, bile acids, and mitochondrial metabolism), inflammatory and fibrogenic processes (collagen fibril organization and metabolism, response to TGFβ), and WNT/β-catenin signaling (Fig. 8C and Suppl. Table 6). The comparison between epitranscriptomic subgroups (EpiT-high vs EpiT-low) in NASH primarily revealed processes associated with collagen biosynthesis, response to TGFβ, and pathways involved in cell adhesion and migration, epithelial-mesenchymal transition phenomena, and angiogenesis (Fig. 8D and Suppl. Table 7).

In view of the key pathogenic role of fibrosis in the progression of NAFLD [5, 10, 30], we evaluated the potential correlation in the expression of epigenetic and epitranscriptomic genes with that of key genes involved in liver extracellular matrix synthesis. We selected the genes coding for fibrillar collagens I, III, and V; interfibrillar collagen VI; and collagen IV, which together with collagen VI is responsible for the capillarization of liver sinusoids, being all of them being upregulated in liver fibrosis [52]. As shown in Suppl. Fig. 5A, there were several epigenetic genes which showed strong correlations with the expression of these collagen genes, including writers, readers, and erasers such as *CBX6*, *CHD3*, *DNMT1*, *EHMT2*, *HDAC7*, *MLLT3*, *PHF19*, *PRDM2*, *PRMT2*, *SMARCA4*, *TP53BP1*, and *ZBTB4*, among others. Although less prominent, we also observed the correlation of collagen gene expression with that of certain epitranscriptomic genes such as *ADARB1*, *APOBEC3G*, *HEMT1*, *IGFBP2*, *PRRC2A*, *TCOF1*, and *TRMT1* (Suppl. Fig. 5B). We also included in the analysis the *ACTA2* and *LRAT* genes, coding for α-smooth muscle actin (αSMA) and lecithin-retinol acyltransferase, which are upregulated and downregulated, respectively, in activated liver extracellular matrix (ECM)-producing cells [9]. Most of the epigenetic and epitranscriptomic genes which expression correlated positively with that of collagen genes also correlated with that of *ACTA2*, but not, or very weakly, with *LRAT* gene expression (Suppl. Fig. 5A and 5B), further emphasizing their association with liver disease progression.

As previously mentioned, NASH is increasingly recognized as a risk factor for HCC development [5, 64]. Given the strong association between our epigenetic and epitranscriptomic gene signatures established in NAFLD patients with key molecular pathways related to disease progression described above, we explored whether these signatures could also be observed in peritumoral and tumoral tissues from NASH-associated HCC [85]. As shown in Suppl. Fig. 6A and 6B, subgroups of patients showing EpiG-low and EpiG-high signatures, as well as EpiT-low and EpiT-high signatures, were found in peritumoral and HCC tissues. In contrast with these observations, the expression of genes within the EpiG and EpiT signatures was uniformly altered in peritumoral tissues from patients developing HCC of viral (HBV) [101] or alcoholic [67] etiologies, and these changes were more pronounced in tumoral tissues (Suppl. Fig. 7A-D).

These observations led us to explore if the expression of these epigenetic and epitranscriptomic gene signatures could be linked to the prognosis of HCC patients. To this end, we analyzed data generated by the TCGA network, and as depicted in Supplementary Fig. 8A, we found that HCC patients in the EpiG-high subgroup had significantly poorer survival than those in the EpiG-med and EpiG-low subgroups. However, this difference was not observed for patients in the three EpiT subclasses (Supplementary Fig. 8B).

### Candidate epigenetic and epitranscriptomic biomarkers and therapeutic targets in NAFLD

**Fig. 9:**
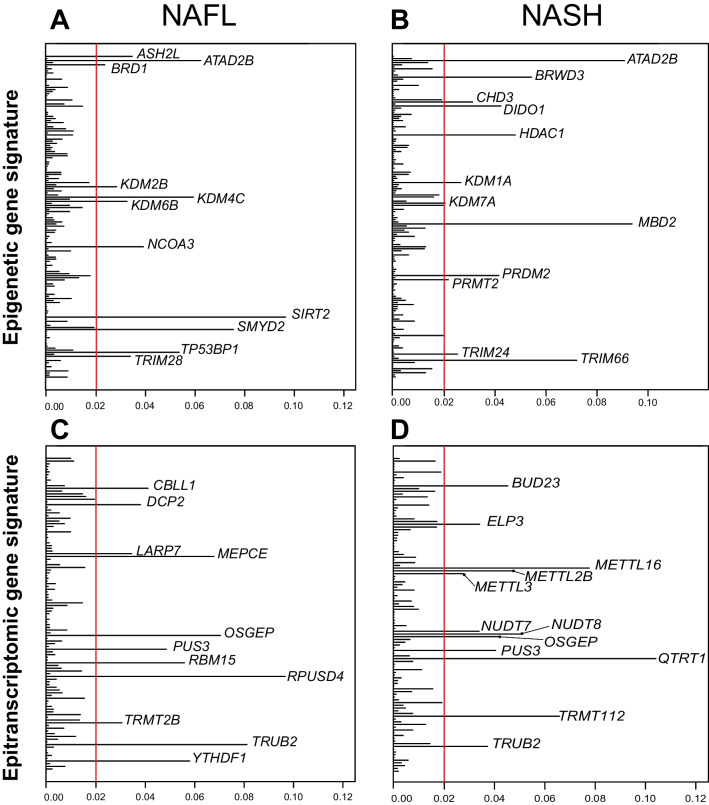
Most relevant genes contributing to the stratification of patients in the low- and high- EpiG and EpiT clusters in NAFL and NASH patients. **A** Most relevant epigenetic genes contributing to the stratification of NAFL patients in the EpiG-low and EpiG-high clusters. **B** Most relevant epigenetic genes contributing to the stratification of NASH patients in the EpiG-low and EPIG-high clusters. **C** Most relevant epitranscriptomic genes contributing to the stratification of NAFL patients in the EpiT-low and EpiT-high clusters. **D** Most relevant epitranscriptomic genes contributing to the stratification of NASH patients in the EpiT-low and EpiT-high clusters. In the loading plot, the scale indicates the contribution to the separation between groups of each gene in a scale from 0 to 1. Selected genes explain at least 2% of the variability between low- and high- groups

Our current findings evidenced marked alterations in the expression of epigenetic and epitranscriptomic genes and their association with liver disease progression. Therefore, among these genes, there could be good candidates to be developed into biomarkers of disease severity and/or targets for pharmacological intervention. With this in mind, we implemented a supervised DAPC analysis for the selection of those genes that accounted for most of the intergroup variability (i.e., those that explained at least 2% of the variability between groups of samples). We performed these DAPC analyses in liver tissues from patients with NAFL and NASH, and for both the epigenetic (Fig. 9A and B) and epitranscriptomic (Fig. 9C and D) gene signatures.

Consistently, the expression of these largest contributors to the epigenetic and epitranscriptomic NASH signatures showed statistically significant upregulation across the EpiG and EpiT subclasses (Suppl. Fig. 9A and B). Interestingly, the expression of most of these epigenetic and epitranscriptomic genes was significantly dysregulated in NASH patients classified according to their fibrosis stage (F0–F4) (Suppl. Fig. 9C). Regarding the epigenetic gene signature, this analysis showed the upregulation of *CHD3* and *PRMT2* and the downregulation of *TRIM24* expression across all fibrosis stages when compared to the F0 group. Also, *DIDO1* and *PRDM2* were upregulated, and *ATAD2B* and *BRWD3* were downregulated in at least one fibrosis stage vs F0. For the epitranscriptomic genes, *QTRT1* and *TRMT112* expression was significantly upregulated across all fibrosis stages, while *TRUB2* was upregulated in F1 to F3 compared to F0, and *METTL3* and *OSGEP* were upregulated in F4 compared to F0 (Suppl. Fig. 9D).

### Differential expression of metabolic genes

**Fig. 10:**
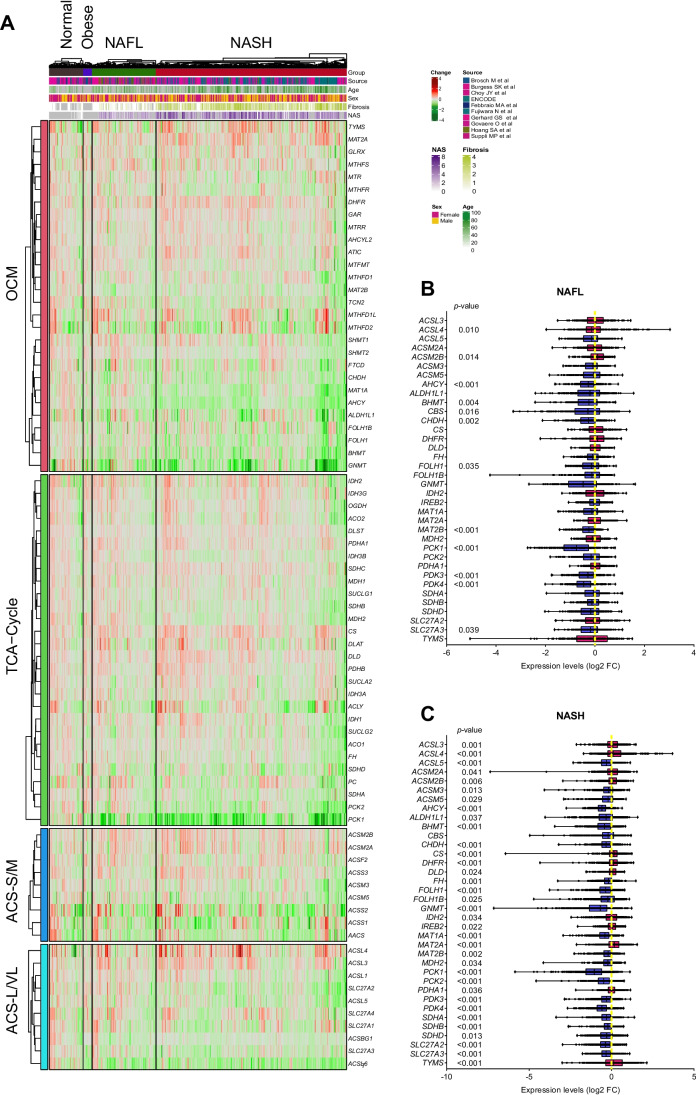
Transcriptomic analysis of genes encoding enzymes involved in the syhthesis and conversion of substrates and cofactors of epigenetic and epitranscriptomic enzymatic reactions in the integrated transcriptomic dataset. **A** Heatmap of the expression of metabolic genes according to liver disease classification. Expression fold change is relative to normal liver. **B** and **C** Expression levels of the indicated metabolic genes genes in NAFL and NASH patients relative to normal liver tissues. Values are expressed as the trimmed mean of *M*-values (TMM) and transformed to log2 fold change versus normal liver. ACS-S/M, short- and medium-chain acyl-coA synthetase; ACS-L/VL, long- and very long-chain acyl-coA synthetase; OCM, one-carbon metabolism; TCA, tricarboxylic acid cycle. *p*-values were obtained from the Kruskal-Wallis test and adjusted by FDR Benjamini and Hochberg correction

Finally, we also examined the expression of 89 genes encoding enzymes belonging to different metabolic pathways such as that of folates and one carbon (OCM) metabolism, tricarboxylic acid cycle (TCA), and the acetyl-CoA synthesis pathway (ACS). These enzymes are implicated in the synthesis and metabolism of cofactors involved in the activity and regulation of most epigenetic and epitranscriptomic reactions (Fig. 1C and D, and Suppl. Table 8) [13, 28, 58]. Unlike what was found for epigenetic and epitranscriptomic genes, a rather homogeneous expression pattern was found across samples within the NAFL and NASH groups (Fig. 10A). Significant changes were observed in the expression of 12 metabolic genes in NAFL when compared to normal liver samples (Fig. 10B), of which 10 were downregulated and 2 were upregulated. Regarding the expression of metabolic enzymes in NASH liver samples, these changes were much more pronounced. The expression of 35 genes involved in all the above-mentioned metabolic pathways was significantly altered when compared to normal liver tissues, with 23 genes being downregulated and 12 upregulated (Fig. 10C).

## Discussion

Weight loss through nutritional intervention and physical activity can improve liver disease in NAFLD, but currently there are no approved therapies to treat this condition. The progression of fibrosis, the deployment of cirrhosis, and the advent of adverse liver-related complications such as decompensation and HCC development are still quite unpredictable [17, 30, 64, 103]. A better understanding of the molecular mechanisms underlying this complex disease is needed to predict and eventually treat these outcomes. Accumulating experimental and clinical evidence indicates that epigenetic and epitranscriptomic alterations occur during NAFLD development [2, 22, 49, 86, 106, 123]. These dynamic processes are shaped by lifestyle, environment, and enduring risk factors, and their persistent influence on gene expression may be linked to metabolic derangement and disease progression in NAFLD [25, 38, 57, 65, 78, 119, 124].

In our study, we combined ten liver transcriptomic datasets including patients at different stages of NAFLD progression together with obese and normal individuals so they can be interrogated as a single transcriptome. After validating the robust integration of the different datasets, we performed a comprehensive study of the expression of epigenetic and epitranscriptomic modifiers, including 20 epigenetic and 6 epitranscriptomic gene families. Our analyses revealed significant variations in their expression patterns across NAFL and NASH patients in comparison with normal liver samples. As could be anticipated, there were more pronounced statistically significant differences in both families of modifiers in patients with NASH. Among the 379 analyzed samples, 108 epigenetic effectors and 40 epitranscriptomic genes exhibited differential expression. Some of these genes have been previously reported to show altered expression in both NAFL and NASH stages, including epigenetic genes such as *DNMT1*, *SIRT1*, and *ZBTB33*, and epitranscriptomic genes like *IGFBP1*. However, for most of these genes, their differential expression in NAFL or NASH had not been previously described. Among them, we found *HAT1*, which codes for a histone acetyl- and succinyltransferase recently reported to be induced in HCC with protumorigenic consequences [114], and *SMYD2*, a histone methyltransferase also induced in HCCs with poor prognosis and just described to be critical for the development of experimental steatosis in mice [107, 113]. *CBX1* and *MPHOSPH8*, epigenetic readers binding methylated lysine residues, were also markedly upregulated in NASH tissues. Although their upregulation in chronic liver disease has not been reported, both have been involved in carcinogenesis, including HCC development in the case of *CBX1* [81, 100]. Similarly, genes coding for epitranscriptomic readers like *YTHDF3* and *YTHDC2* and epitranscriptomic writers such as *RNMT*, *METTL5*, *TRNMT10C*, and *PUS7L* were upregulated in NASH tissues and are known to be involved as well in hepatocarcinogenesis [45, 60, 62, 88, 98, 110].

Perhaps most interestingly, we also observed that within the NAFL and NASH cohorts, distinct patterns of expression of both epigenetic and epitranscriptomic genes were apparent among patients. Through hierarchical clustering, we identified three subtypes of patients based on their high, intermediate, or low expression of these two classes of genes. Interestingly, correlation analyses with clinico-pathological information showed that the EpiT-high and the EpiG-high subtypes, but specially the latter, encompassed the highest proportion of patients with advanced fibrosis (F4, cirrhosis). A particularly strong correlation between the expression of epigenetic effectors and collagen genes was evidenced, indicating that these genes are likely involved in disease progression, as previously suggested [8, 75]. This notion was reinforced when we performed functional analyses of the differentially expressed genes between each EpiG and EpiT subtype within NAFL and NASH cohorts. At the NAFL stage, the EpiT-high subgroup presented more active pathways involved in harmful events such as DNA damage, cell death, and inflammatory activation and signaling. Moreover, those patients within the EpiT-high signature also showed marked downregulation in gene sets related to aerobic respiration, mitochondrial function, fatty acid metabolism, and response to oxidative stress and insulin. Although changes in the expression of specific epitranscriptomic and epigenetic genes may have important consequences for disease development, globally, it appears that epitranscriptomic mechanisms could play adaptive and eventually pathogenic roles early in NAFLD progression. This idea fits well with the very dynamic nature of epitranscriptomic modifications, such as N6-methyladenosine, and their key role in the acute regulation of metabolic genes [106, 121]. Moreover, in line with our findings, a recent study found no significant relationship between the expression of N6-methyladenosine regulators and liver fibrosis in NAFLD patients [22]. Growing evidence indicates that epigenetic changes, particularly histones and DNA methylation, can even persist after removal of harmful exposures such as food restriction or bad dietary habits, a phenomenon known as maladaptive epigenetic memory [21, 22, 57, 83]. Therefore, certain persistently dysregulated epigenetic genes could be responsible for this long-term epigenetic memory contributing to disease progression. In this regard, in the NASH stage, the comparison between EpiG-low and EpiG-high revealed a clear stratification of patients with strong alterations in many important biological pathways mostly associated with lipid and steroid metabolism, ribosomal processes, inflammation, and fibrogenesis.

The magnitude of gene expression variation between different biological conditions does not necessarily relate to a more prominent functional role for a specific gene. Nevertheless, given the numerous epigenetic and epitranscriptomic genes differentially expressed between the EpiG and EpiT subclasses of patients, we performed an unbiassed analysis to identify those that contributed most to this stratification. Among the upregulated epigenetic genes were the reader and chromatin remodeller *CHD3* [3], the methylated lysine reader *DIDO1*, the arginine methyltransferase *PRMT2*, and the lysine methyltransferase *PRDM2*, not previously described in NASH. Noteworthy, *PRMT2* was recently reported to be induced and contribute to HCC tumorigenesis [39], *DIDO1* has been involved in numerous types of tumors [16], and *PRDM2* is a tumor suppressor epigenetically repressed in HCC [120]. Conversely, the acetylated histone reader *TRIM24* was downregulated along fibrosis progression. Interestingly, *TRIM24* is known to repress hepatic lipid accumulation and fibrosis in the murine liver [43]; however, its expression in human NAFLD has not been reported before. Among epitranscriptomic genes, we found *QTRT*, a queuine tRNA ribosyltransferase known to be induced in lung cancer [66], and *TRUB2*, a pseudouridine synthase essential for mitochondrial protein synthesis [6]. In this set of genes, we also identified *TRMT112* which codes for a methyltransferase known to be induced in different cancers including HCC [112]. Most interestingly, TRMT112 was recently described to form an N6-methyladenosine methyltransferase complex with METTL5, which we also found upregulated in NASH, that remodels fatty acid metabolism and promotes HCC tumorigenesis [84].

In this study, we have identified for the first time numerous epigenetic and epitranscriptomic genes dysregulated along the course of human NAFLD. Interestingly, being NAFLD primarily a metabolic condition, the pathophysiological roles described in the literature for most of these genes are related to carcinogenic processes, including HCC development [15, 29, 106, 123]. Indeed, NAFLD is an emerging risk factor for liver cancer, particularly in patients with type 2 diabetes [64]. While just changes in the expression of epigenetic and epitranscriptomic effectors are not likely to be tumorigenic per se, these alterations may indeed facilitate metabolic rewiring and pave the way for neoplastic transformation triggered by NAFLD-HCC driving mutations [85]. Supporting this notion, there is already experimental evidence showing that drug-mediated inhibition of epigenetic reprogramming can improve NASH and fibrosis progression [73] and prevent NASH-associated HCC development [51], illustrating the therapeutic potential of targeting these pathways. Importantly, when we analyzed the TCGA HCC cohort, we observed that patients within the EpiG-high subclass had significantly lower survival than those in the EpiG-med or EpiG-low subclasses. This finding suggests that the epigenetic genes comprised in a signature that defines advanced NAFLD stages may also be involved in HCC progression. Nevertheless, it would be interesting to test the predictive capacity of this EpiG signature in a selected cohort of NASH-related HCC patients.

Epigenetic and epitranscriptomic mechanisms are complex and intertwined, and fluctuations in the expression of these effectors are likely to influence the course of NAFLD progression. However, gene expression regulation at the chromatin and epitranscriptomic levels involves another layer of complexity linked to the intrinsic enzymatic nature of these processes. As previously mentioned, epigenetic and epitranscriptomic writers and erasers utilize a range of metabolites as substrates and cofactors (Fig. 1C). For chromatin regulatory mechanisms, this interaction has proven so relevant that epigenetic processes can even act as sensors of the activity of central metabolic pathways, including pyruvate metabolism and the TCA cycle, acetyl-CoA, NAD^+^, FAD, S-adenosylmethionine (SAM)/OCM, and energetic metabolism (AMP/ATP), and in turn can regulate the expression of metabolism-related genes [13, 21, 58]. Indeed, the intracellular, and even intranuclear, levels of metabolites that behave as substrates or inhibitors of epigenetic and epitranscriptomic reactions, such as SAM and S-adenosylhomocysteine (SAH) for methylation/demethylation; α-ketoglutarate (α-KG), fumarate, and succinate for demethylation; and acetyl-CoA and NAD for acetylation/deacetylation, strongly influence the homeostasis of these processes [13, 58]. Given the profound metabolic alterations occurring in NAFLD, changes in the levels of these metabolites are likely to happen, albeit their direct measurement in human liver tissues is quite challenging [40, 95]. Our transcriptomic analyses revealed profound alterations in the expression of genes coding for key enzymes in the metabolism of epigenetic and epitranscriptomic substrates and cofactors (Fig. 1D). These included acyl-CoA synthetases involved in fatty acid metabolism and acetyl-CoA synthesis such as *ACSL4*, which is also markedly induced in HCC [89], the key TCA cycle enzyme citrate synthase (*CS*), and the gluconeogenic rate-limiting enzyme *PCK1*, also relevant for acetyl-CoA availability [115]. Interestingly, we also observed a marked reduction in the expression of *SDHA*, consistent with observations in murine NASH models [63] and with the increased levels of succinate found in human NASH liver tissues [95]. Importantly, succinate can compete with α-KG and inhibit enzymes involved in histone, DNA, and RNA demethylation [13, 59]. Noteworthy, we found significant alterations in the expression of genes involved in folate metabolism and OCM. Low serum folate levels are consistently found in NAFLD patients; however, the expression of enzymes involved in folate metabolism has not been examined in the liver of these patients [24, 96]. We found induced and repressed levels of *DHFR* and *FOLH1* expression, respectively, in NASH patients. Impaired folate metabolism may affect the synthesis of SAM from homocysteine, and enzymes involved in the metabolism of this non-proteinogenic amino acid were also significantly affected in these patients [96]. Expression of *BHMT*, an enzyme that converts homocysteine back to methionine using betaine as a methyl donor, was significantly repressed in NASH, as was the expression of *CHDH*, the enzyme that synthesizes betaine from choline [80]. Choline deficiency is well known to promote NASH in murine models, and choline metabolism is impaired at different levels in NASH patients [24]. Interestingly, *BHMT* knockout in mice results in liver SAM depletion along with NAFLD and HCC development [102]. We confirmed the downregulation of *GNMT* and *MAT1A* expression in NASH [68, 77], key enzymes in the consumption and synthesis of SAM in the liver, respectively, and also found a marked reduction in *ACHY* expression, which codes for the enzyme in charge of metabolizing SAH into adenosine and homocysteine [80]. Experimental studies in genetically modified mice and diet-induced NAFLD suggest that alterations in *GNMT*, *MAT1A*, and *ACHY* expression can contribute to NASH development in humans [72, 80, 87]. These transcriptional alterations in OCM-related genes can significantly modify on the hepatocellular SAM/SAH ratio, and therefore impact on numerous epigenetic and epitranscriptomic methylation reactions [13].

The changes in metabolic gene expression summarized above may affect the levels of key metabolites in epigenetic and epitranscriptomic reactions; however, there is little information available on the actual levels of these molecules in healthy liver and NAFLD. Emerging technologies such as spatial metabolomics will be crucial in providing this information [94]. Nevertheless, recent molecular and genetic studies reveal that the function of many epigenetic enzymes extends beyond their catalytic activity [76]. This “epigenetic moonlighting” must be taken into account when evaluating the contribution of these effectors to the pathogenesis of NAFLD, and also at the time of designing potential therapeutic interventions which perhaps would need also to look beyond their enzymatic inhibition [23].

In this study, we have provided a comprehensive overview of the expression of epigenetic and epitranscriptomic genes in NAFLD. Of course, it will be important to validate these transcriptional changes at the protein level, and to identify the key target genes downstream these epi-regulators. Likewise, it will be very interesting to understand the upstream mechanisms that control the expression of epigenetic and epitranscriptomic genes in the context of NAFLD. These are likely to be multifarious, and involve signaling pathways associated with the prevalent pro-inflammatory and lipotoxic environment that characterizes NAFLD. Interestingly, the presence of the PNPLA3 I148M variant, strongly associated with NASH progression [27], has been recently reported to trigger potent pro-inflammatory signaling in cultured hepatocytes [82]. Nonetheless, our work may help in the elucidation of the pathogenic mechanisms of this complex disease, and also for the identification of pathways contributing to HCC development in this condition.

### Supplementary information

ESM 1 (PDF 14.1 mb)

ESM 2 (XLSX 416 kb)

## Abbreviations

NAFLD: Non-alcoholic fatty liver disease
NAFL: Non-alcoholic fatty liver
NASH: Non-alcoholic steatohepatitis
HCC: Hepatocellular carcinoma
SRA: Sequence Read Archive
TMM: Trimmed mean of *M*-values
PCA: Principal component analysis
UMAP: Uniform manifold approximation and projection
*t*-SNE: *t*-distributed stochastic neighbor embedding analysis
PC: Principal components
GSVA: Gene set variation analysis
ssGSEA: Single-sample gene set enrichment analysis
GLM: Generalized linear model
FDR: False discovery rate
GSEA: Gene set enrichment analysis
EpiG: Epigenetic
EpiT: Epitranscriptomic
GO: Gene Ontology
DEGs: Differentially expressed genes
ECM: Extracellular matrix
DAPC: Discriminant analysis of principal components
OCM: One carbon metabolism
TCA: Tricarboxylic acid cycle
