# Supplementary material for: Comprehensive analysis of epigenetic and epitranscriptomic genes’ expression in human NAFLD
Source: J Physiol Biochem. 2023 Aug 25;79(4):901–24. doi: 10.1007/s13105-023-00976-y (PMC10636027; doi:10.1007/s13105-023-00976-y)
Supplement: Supplementary file 1 — (PDF 14.1 mb) [file 13105_2023_976_MOESM1_ESM.zip › 13105_2023_976_MOESM1_ESM/Herranz et al. Legends to Supplementary Figures and Tables Revised_ESM.docx]

**Legends to the Supplementary Figures**

**Supplementary Figure 1.** Complementary gene expression analyses for the validation of the biological relevance of the integrative transcriptomic data from liver samples. **A**: Additional genes from the previously published gene signatures of NAFLD in the integrative transcriptomic data according to liver disease classification (Normal, obese, NAFL, and NASH). **B**: Gene expression levels of the previously published gene signatures of NAFLD and a selection of previously published confident NAFLD pathology markers grouped according to fibrosis stage classification (F0-F4), expressed as the trimmed mean of M-values (TMM). *P*-values were obtained from the Kruskal-Wallis test and adjusted by FDR Benjamini and Hochberg correction. Values of *p*<0.05 were considered statistically significant.

**Supplementary Figure 2.** Heatmap of the expression of non-conventional epigenetic genes grouped in families according to liver disease classification (normal liver, liver in obese patients, NAFL and NASH). Expression fold change is compared with normal liver.

**Supplementary Figure 3.** Gene expression levels of a selection of **A** epigenetic and **B** epitranscriptomic genes. Transcriptomic data are expressed as the trimmed mean of M-values (TMM) and grouped according to liver disease classification (Normal, obese, NAFL and NASH). *p*-values were obtained from the Kruskal-Wallis test and adjusted by FDR Benjamini and Hochberg correction. Values of *p*<0.05 were considered statistically significant.

**Supplementary Figure 4.** Distribution of male and female NASH patients according to the expression of the EpiG and EpiT gene signatures. According to the EpiG signature, among female patients there were 118 EpiG-low, 135 EpiG-med and 33 EpiG-high. For male patients, there were 110 EpiG-low, 145 EpiG-med and 38 EpiG-high. Classification of female patients according to the EpiT signature resulted in 73 EpiT-low, 78 EpiT-med and 135 EpiT-high, and for male patients there were 76 EpiT-low, 77 EpiT-med and 140 EpiT-high. No statistically significant differences were observed.

**Supplementary Figure 5.** Gene expression correlation between fibrosis-related genes and epigenetic and epitranscriptomic genes. Correlation matrix, plotted in a correlogram, obtained by applying a Pearson analysis to the total of **A** epigenetic genes or **B** epitranscriptomic genes and a selection of genes involved in fibrosis. Markers of extracellular matrix producing cells activation *ACTA* (upregulated) and *LRAT* (downregulated) were also included. Dark blue corresponds to +1 *r*-values, indicating a strong positive linear correlation between the genes; white corresponds to 0 *r*-values, indicating that no correlation exists; dark red corresponds to -1 *r*-values, indicating a strong negative linear correlation. The magnitude of the correlation, when present, is indicated by the size of the dot. Only the significant correlations (*p*<0.05) were plotted.

**Supplementary Figure 6.** Heatmaps showing the expression of: **A** EpiG gene signature and **B** EpiT gene signature in normal liver, NASH, peritumoral tissues and tumoral tissues developed on a NASH background [85]. Expression fold change is compared with normal livers.

**Supplementary Figure 7.** Heatmaps showing the expression of: **A** EpiG gene signature and **B** EpiT gene signature in normal liver, peritumoral tissues and tumoral tissues developed on an HBV-infection background [101]; **C** EpiG signature and **D** EpiT gene signature in normal liver, peritumoral tissues and tumoral tissues from patients that developed HCC of due to alcohol abuse (72%), HCV infection (15%) and HBV infection (15%)[67]. Expression fold change is compared with normal livers.

**Supplementary Figure 8.** Kaplan-Meier’s plots of patients’ survival according to the expression of the **A** EpiG and **B** EpiT gene expression signatures. Transcriptomic and patients’ outcome information were obtained from data generated by the TCGA research network (https://www.cancer.gov/tcga).

**Supplementary Figure 9.** Expression of the genes that contributed most to define the epigenetic and epitranscriptomic NASH signatures. **A** Expression of the indicated epigenetic genes in the EpiG-low, EpiG-medium and EpiG-high subclasses in NASH patients. **B** Expression of the indicated epitranscriptomic genes in the EpiT-low, EpiT-medium and EpiT-high subclasses in NASH patients. **C** Expression of the above indicated epigenetic genes in NASH liver tissues grouped acording to their fibrosis stage (F0-F4). **D** Expression of the above indicated epitranscriptomic genes in NASH liver tissues grouped acording to their fibrosis stage (F0-F4). Gene expression levels are expressed as the trimmed mean of M-values (TMM). *p*-values were obtained from the Kruskal-Wallis test and adjusted by FDR Benjamini and Hochberg correction. Values of *p*<0.05 were considered statistically significant.

**Legends to the Supplementary Tables**

**Supplementary Table 1**. Selected epigenetic factors classified as writers, erasers, or readers. Genes were categorized by their enzymatic activity on DNA and histones, or their interactions with these molecules. The first original publication, to our knowledge, demonstrating their activity is indicated. Out of 419 selected epigenetic genes, the expression of 379 was detected in the RNAseq analyses and 40 were not detected, as indicated.

**Supplementary Table 2.** Selected epitranscriptomic factors classified as writers, erasers, or readers. Genes were categorized by their enzymatic activity on RNA, or their interaction with this molecule. The first original publication, to our knowledge, demonstrating their activity is indicated. Out of 137 selected epitranscriptomic genes, the expression of 128 was detected in the RNA-seq analyses and 9 were not detected, as indicated.

**Supplementary Table 3.** List of genes included in the epigenetic and epitranscriptomic gene signatures.

**Supplementary Table 4.** Pathway analysis on the differentially expressed genes between EpiG-low and EpiG-high subclasses in samples from patients with NAFL.

**Supplementary Table 5.** Pathway analysis on the differentially expressed genes between EpiT-low and EpiT-high subclasses in samples from patients with NAFL.

**Supplementary Table 6.** Pathway analysis on the differentially expressed genes between EpiG-low and EpiG-high subclasses in samples from patients with NASH.

**Supplementary Table 7.** Pathway analysis on the differentially expressed genes between EpiT-low and EpiT-high subclasses in samples from patients with NASH.

**Supplementary Table 8.** Selected metabolic enzymes and metabolites classified by their involvement in a process of metabolism
