# Supplementary material for: Comprehensive analysis of epigenetic and epitranscriptomic genes’ expression in human NAFLD
Source: J Physiol Biochem. 2023 Aug 25;79(4):901–24. doi: 10.1007/s13105-023-00976-y (PMC10636027; doi:10.1007/s13105-023-00976-y)
Supplement: Supplementary file 1 — (PDF 14.1 mb) [file 13105_2023_976_MOESM1_ESM.zip › 13105_2023_976_MOESM1_ESM/Suppl. Fig. 9_ESM.pdf]

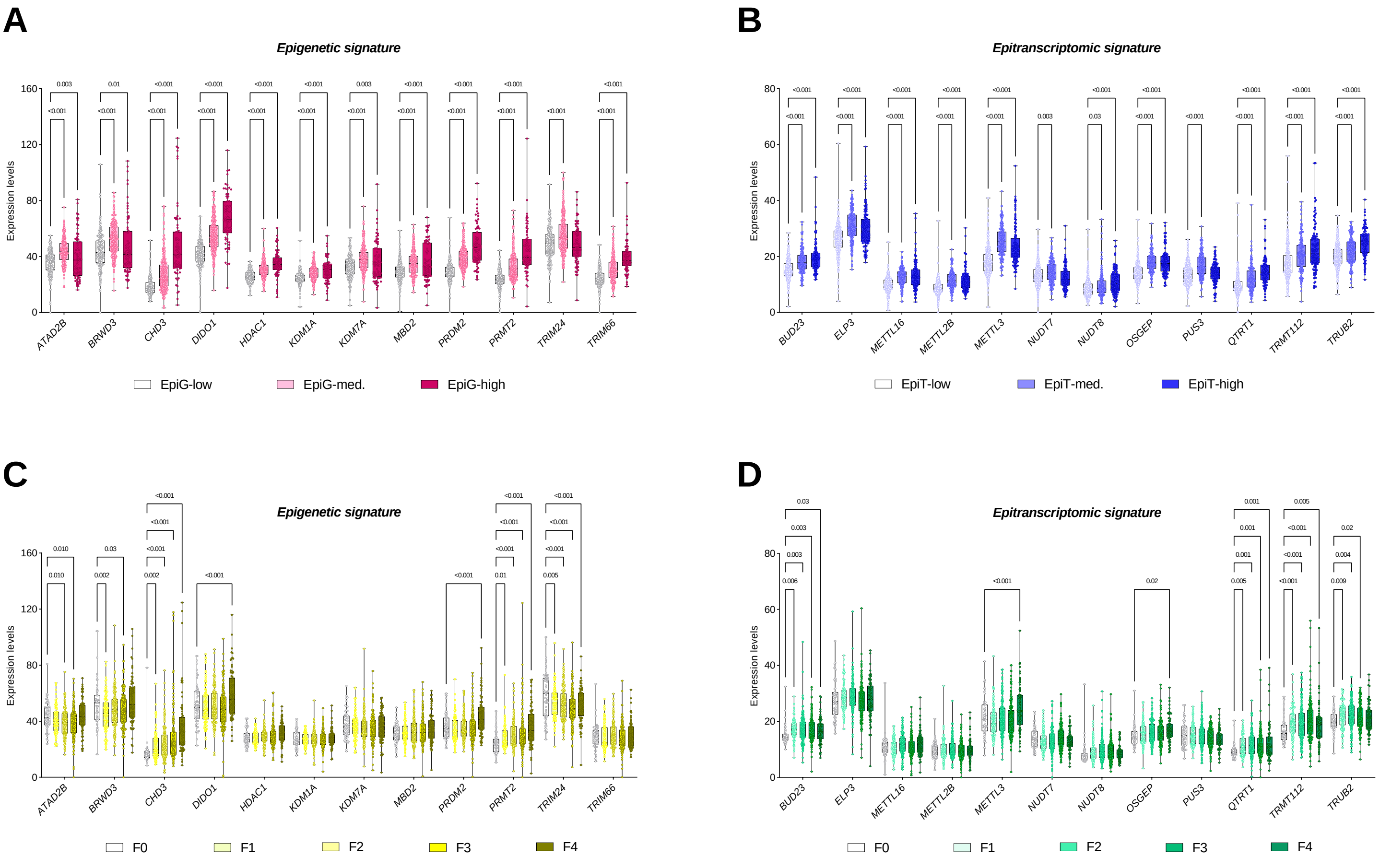

A
Epigenetic signature
Expression levels
0
40
80
120
160
0.003
0.01
<0.001
<0.001
<0.001
<0.001
0.003
<0.001
<0.001
<0.001
<0.001
<0.001
<0.001
<0.001
<0.001
<0.001
<0.001
<0.001
<0.001
<0.001
<0.001
<0.001
<0.001
<0.001
ATAD2B
BRWD3
CHD3
DIDO1
HDAC1
KDM1A
KDM7A
MBD2
PRDM2
PRMT2
TRIM24
TRIM66
EpiG-low
EpiG-med.
EpiG-high
B
Epitranscriptomic signature
Expression levels
0
20
40
60
80
<0.001
<0.001
<0.001
<0.001
<0.001
<0.001
<0.001
<0.001
<0.001
<0.001
<0.001
<0.001
<0.001
<0.001
<0.001
0.003
0.03
<0.001
<0.001
<0.001
<0.001
<0.001
BUD23
ELP3
METTL16
METTL2B
METTL3
NUDT7
NUDT8
OSGEP
PUS3
QTRT1
TRMT112
TRUB2
EpiT-low
EpiT-med.
EpiT-high
C
Epigenetic signature
Expression levels
0
40
80
120
160
0.010
0.010
0.03
0.002
<0.001
<0.001
<0.001
0.002
<0.001
<0.001
<0.001
<0.001
<0.001
0.01
<0.001
<0.001
<0.001
0.005
ATAD2B
BRWD3
CHD3
DIDO1
HDAC1
KDM1A
KDM7A
MBD2
PRDM2
PRMT2
TRIM24
TRIM66
F0
F1
F2
F3
F4
D
Epitranscriptomic signature
Expression levels
0
20
40
60
80
0.03
0.003
0.003
0.006
<0.001
0.02
0.001
0.001
0.001
0.005
0.005
<0.001
<0.001
<0.001
0.02
0.004
0.009
BUD23
ELP3
METTL16
METTL2B
METTL3
NUDT7
NUDT8
OSGEP
PUS3
QTRT1
TRMT112
TRUB2
F0
F1
F2
F3
F4
