# Supplementary figures and images for: Comprehensive analysis of epigenetic and epitranscriptomic genes’ expression in human NAFLD
Source: J Physiol Biochem. 2023 Aug 25;79(4):901–24. doi: 10.1007/s13105-023-00976-y (PMC10636027; doi:10.1007/s13105-023-00976-y)

**A**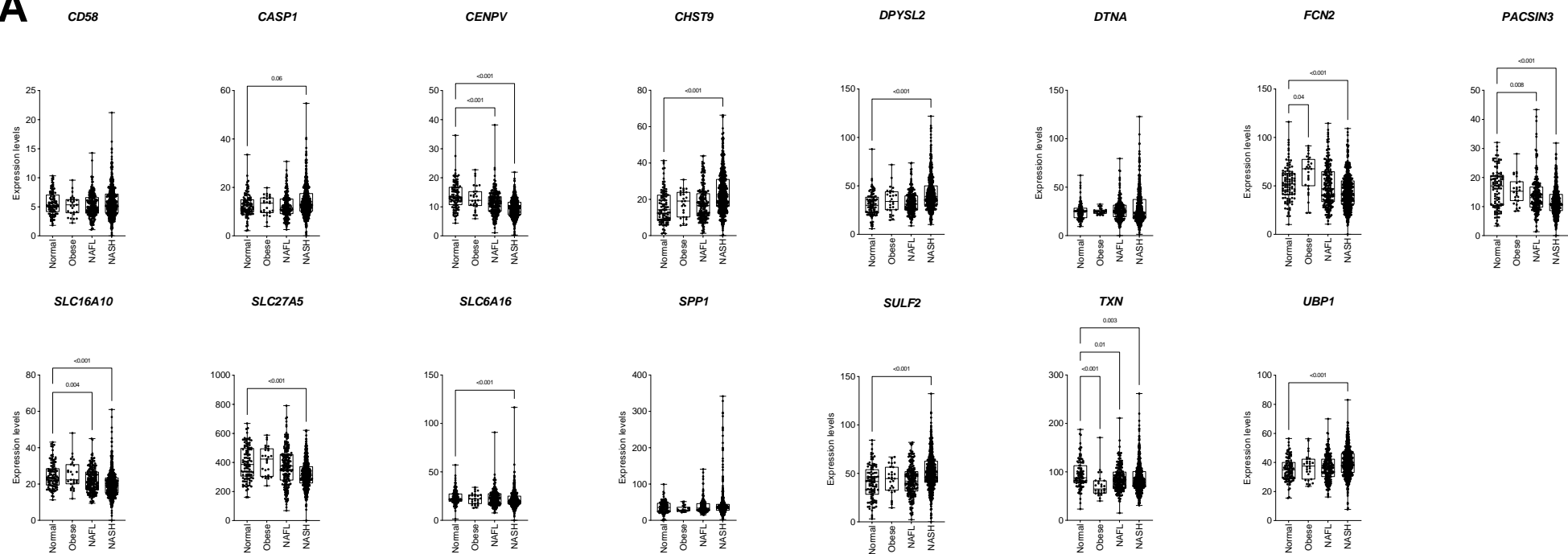**B**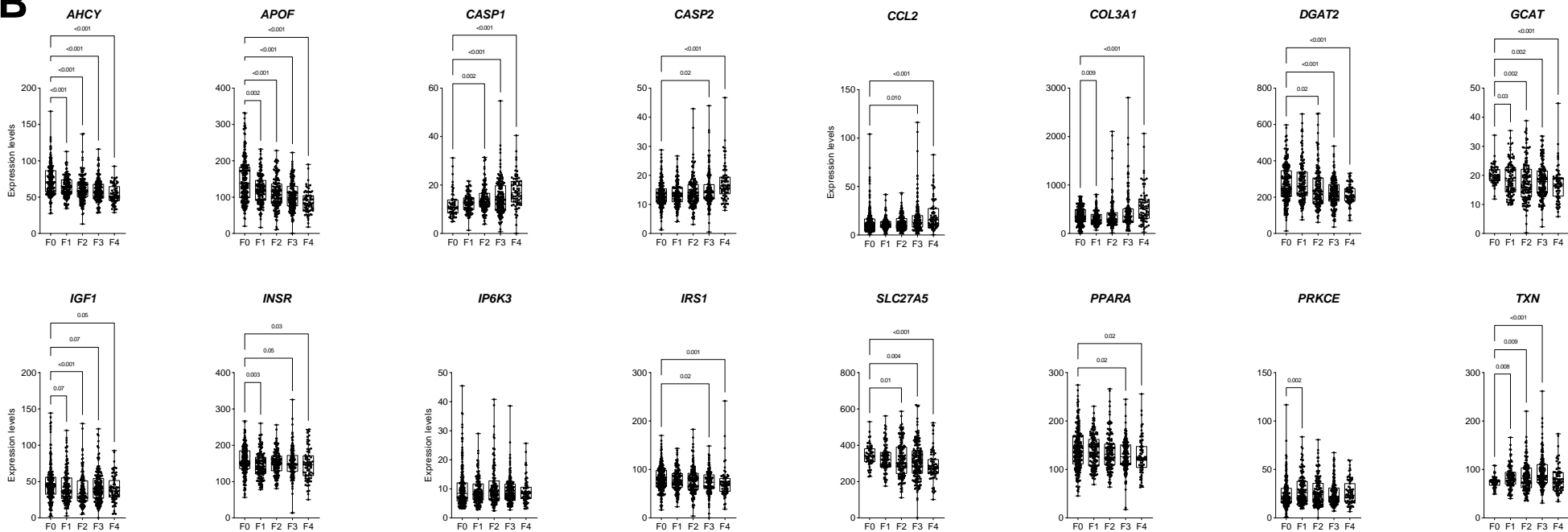

Supplement: Supplementary file 1 — (PDF 14.1 mb) [file 13105_2023_976_MOESM1_ESM.zip › 13105_2023_976_MOESM1_ESM/Suppl. Fig. 1_ESM.pdf]

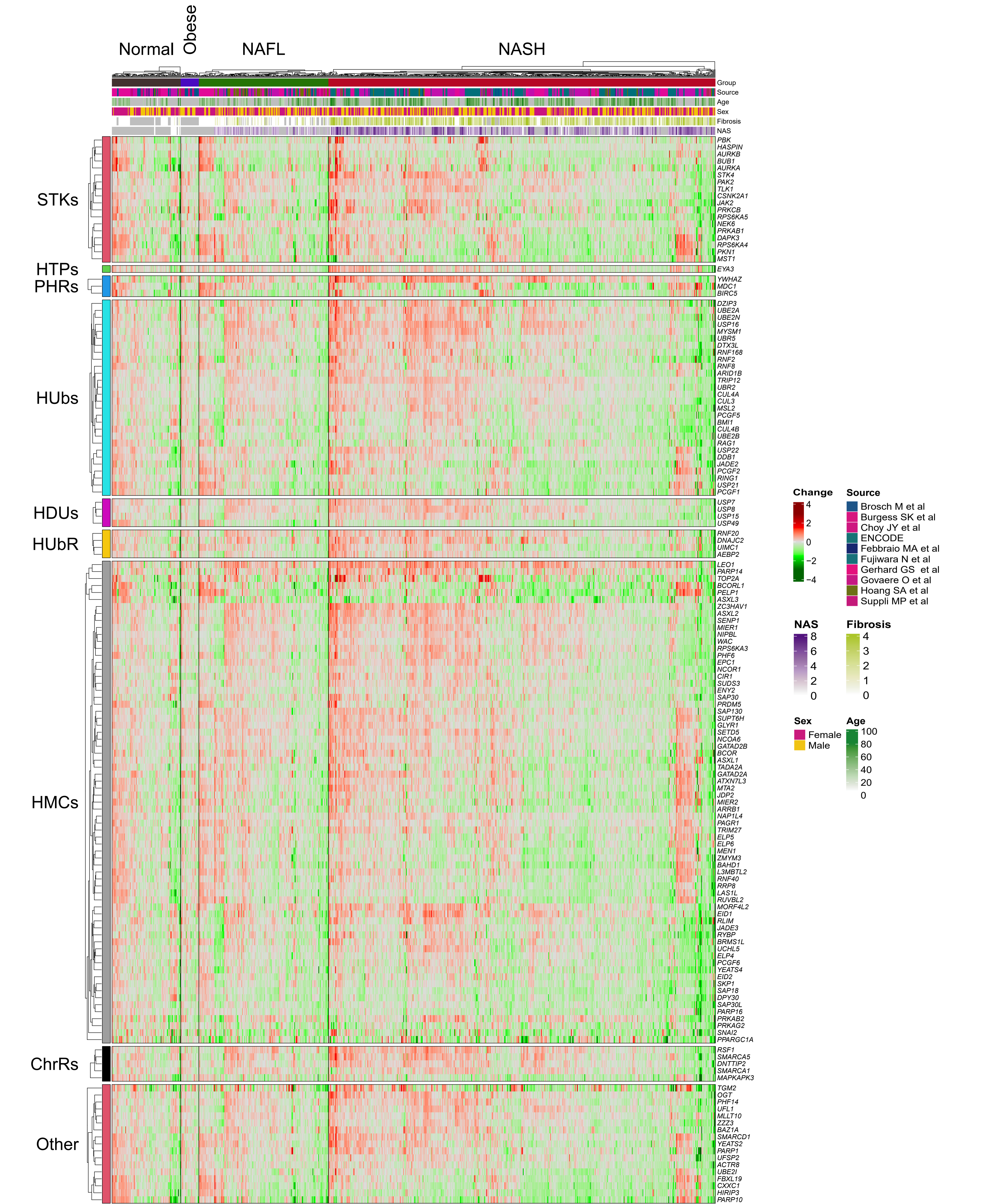

Supplement: Supplementary file 1 — (PDF 14.1 mb) [file 13105_2023_976_MOESM1_ESM.zip › 13105_2023_976_MOESM1_ESM/Suppl. Fig. 2_ESM.pdf]

**A**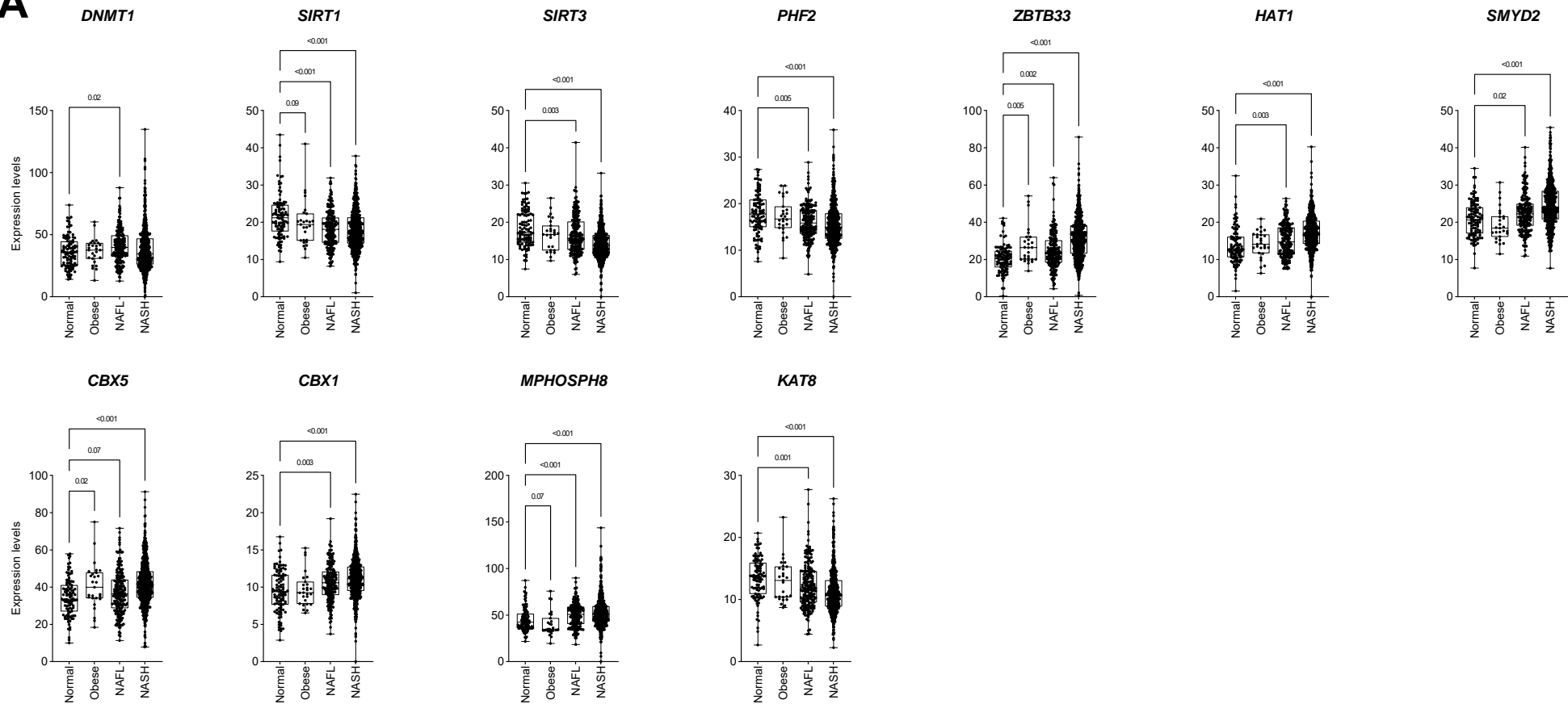**B**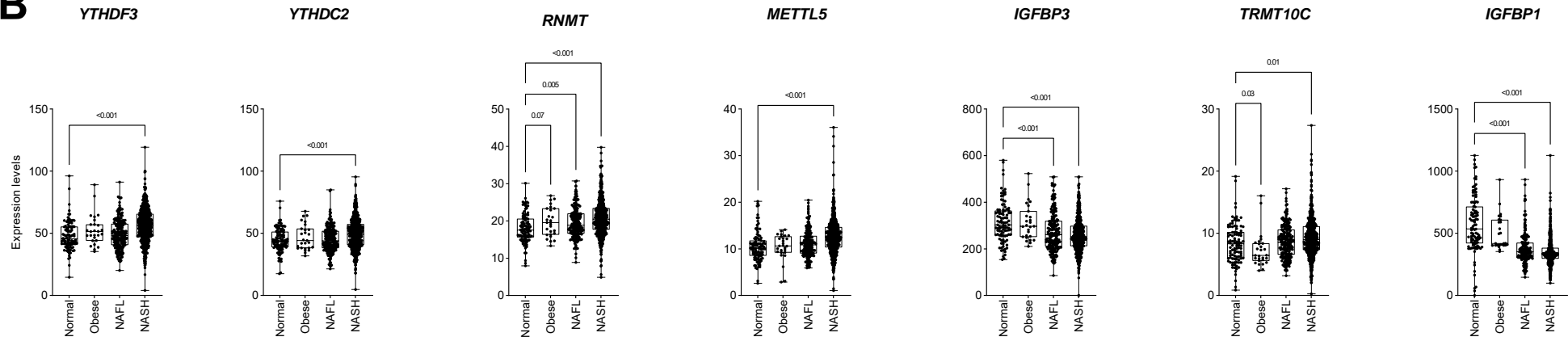

Supplement: Supplementary file 1 — (PDF 14.1 mb) [file 13105_2023_976_MOESM1_ESM.zip › 13105_2023_976_MOESM1_ESM/Suppl. Fig. 3_ESM.pdf]

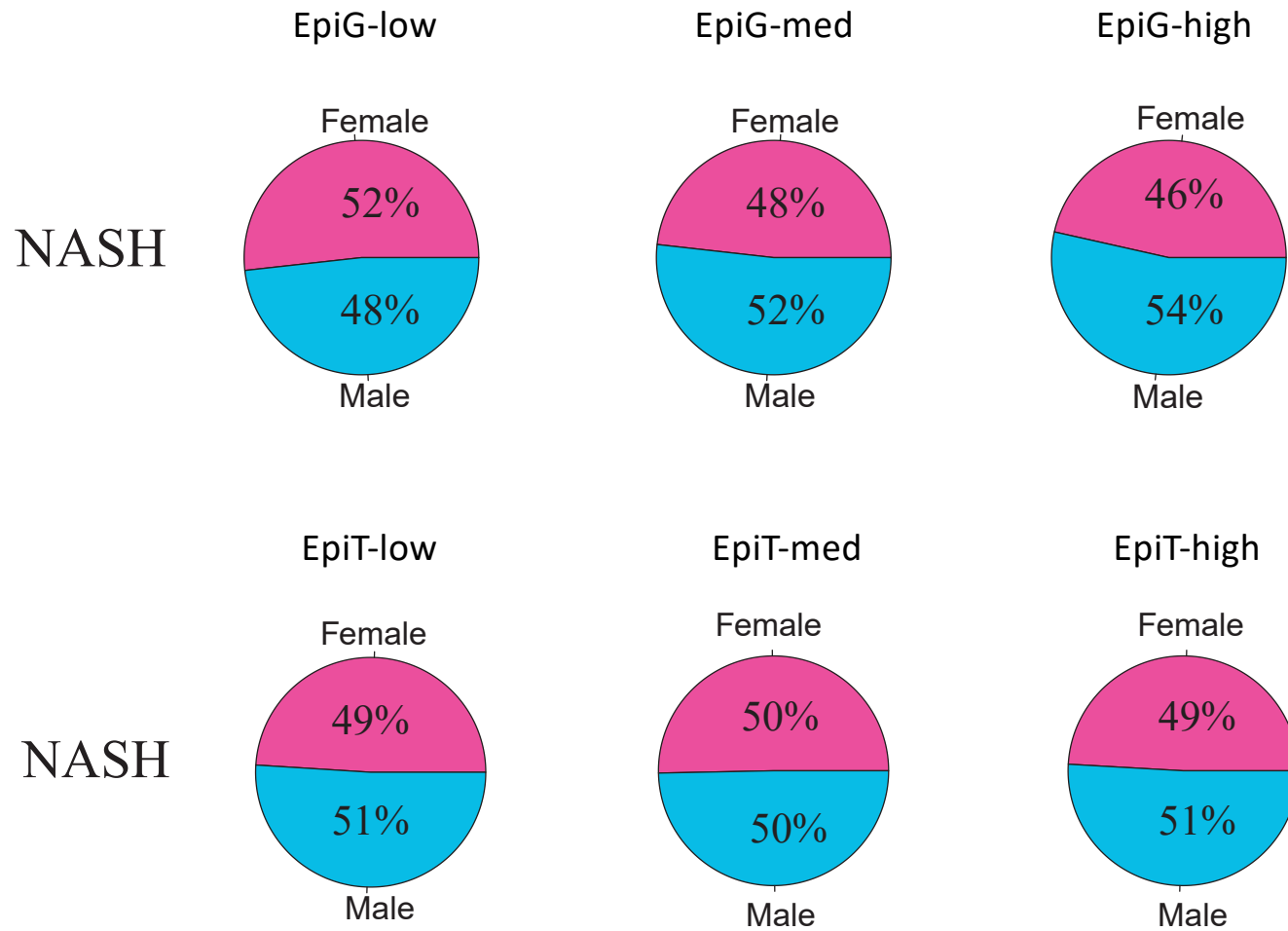

Supplement: Supplementary file 1 — (PDF 14.1 mb) [file 13105_2023_976_MOESM1_ESM.zip › 13105_2023_976_MOESM1_ESM/Suppl. Fig. 4_ESM.pdf]

A

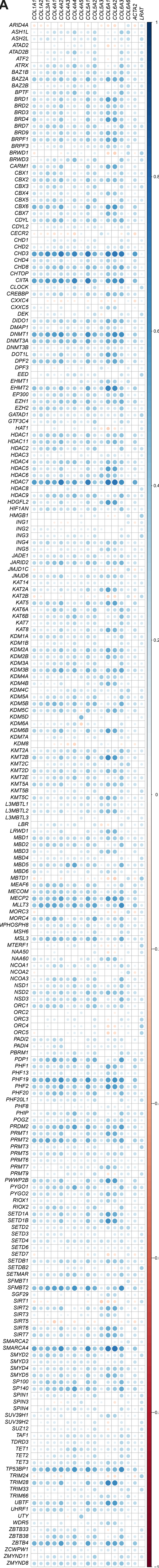

B

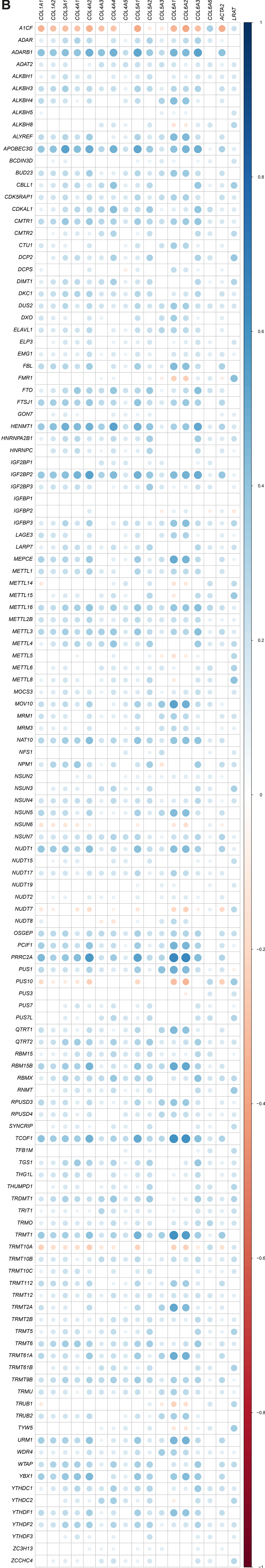

Supplement: Supplementary file 1 — (PDF 14.1 mb) [file 13105_2023_976_MOESM1_ESM.zip › 13105_2023_976_MOESM1_ESM/Suppl. Fig. 5_ESM.pdf]

**A**

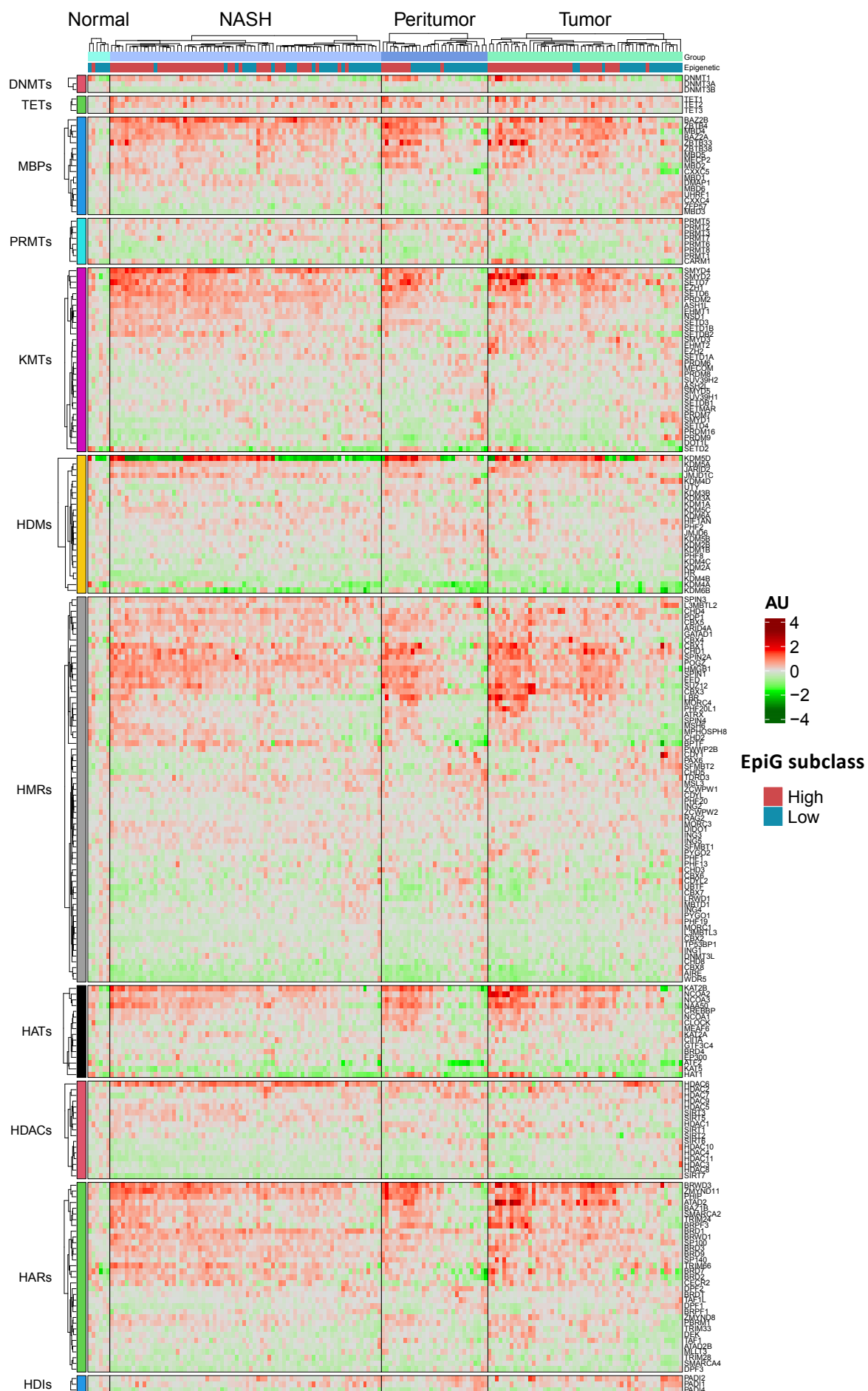

Supplement: Supplementary file 1 — (PDF 14.1 mb) [file 13105_2023_976_MOESM1_ESM.zip › 13105_2023_976_MOESM1_ESM/Suppl. Fig. 6A_ESM.pdf]

**B**

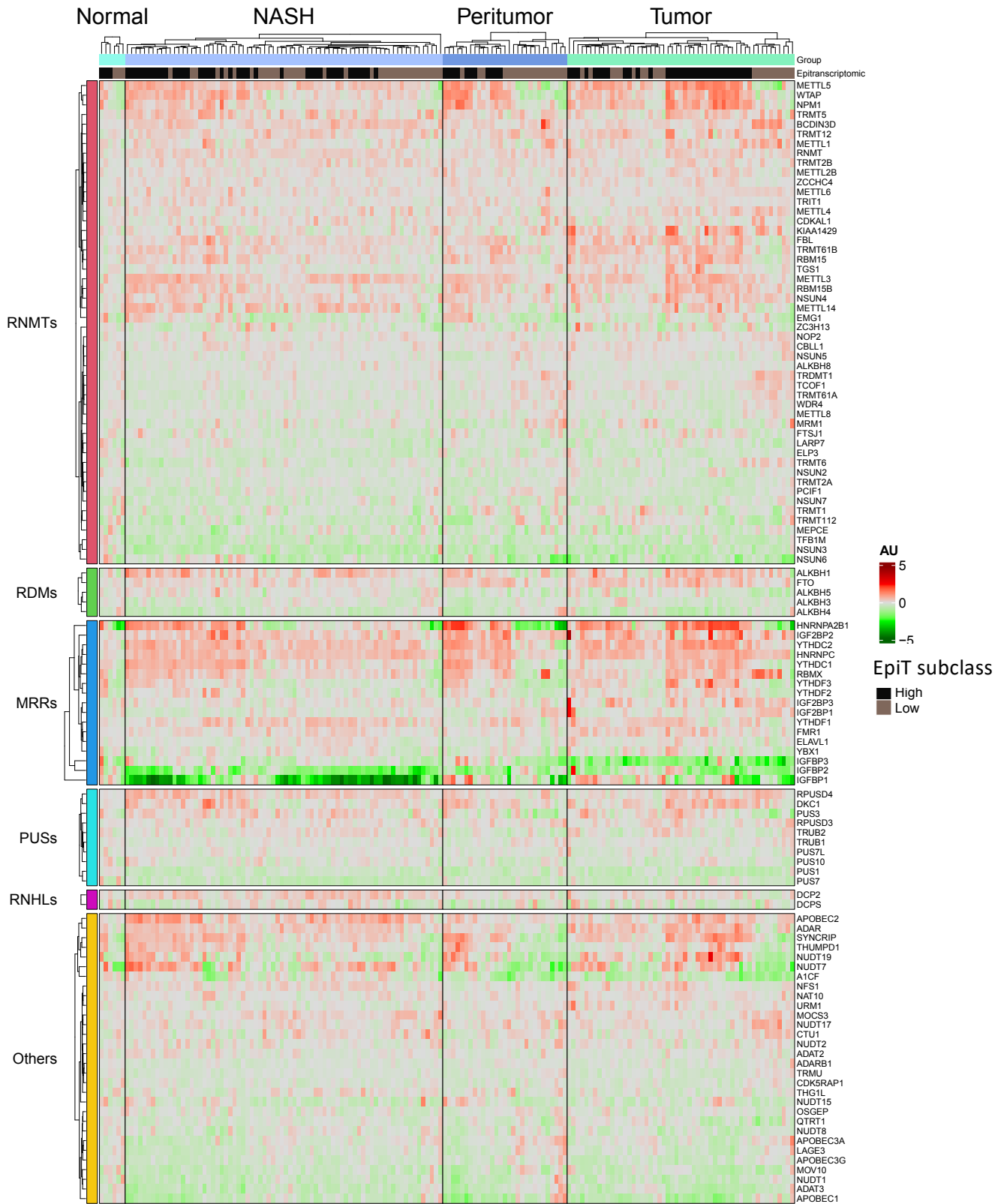

Supplement: Supplementary file 1 — (PDF 14.1 mb) [file 13105_2023_976_MOESM1_ESM.zip › 13105_2023_976_MOESM1_ESM/Suppl. Fig. 6B_ESM.pdf]

**A**

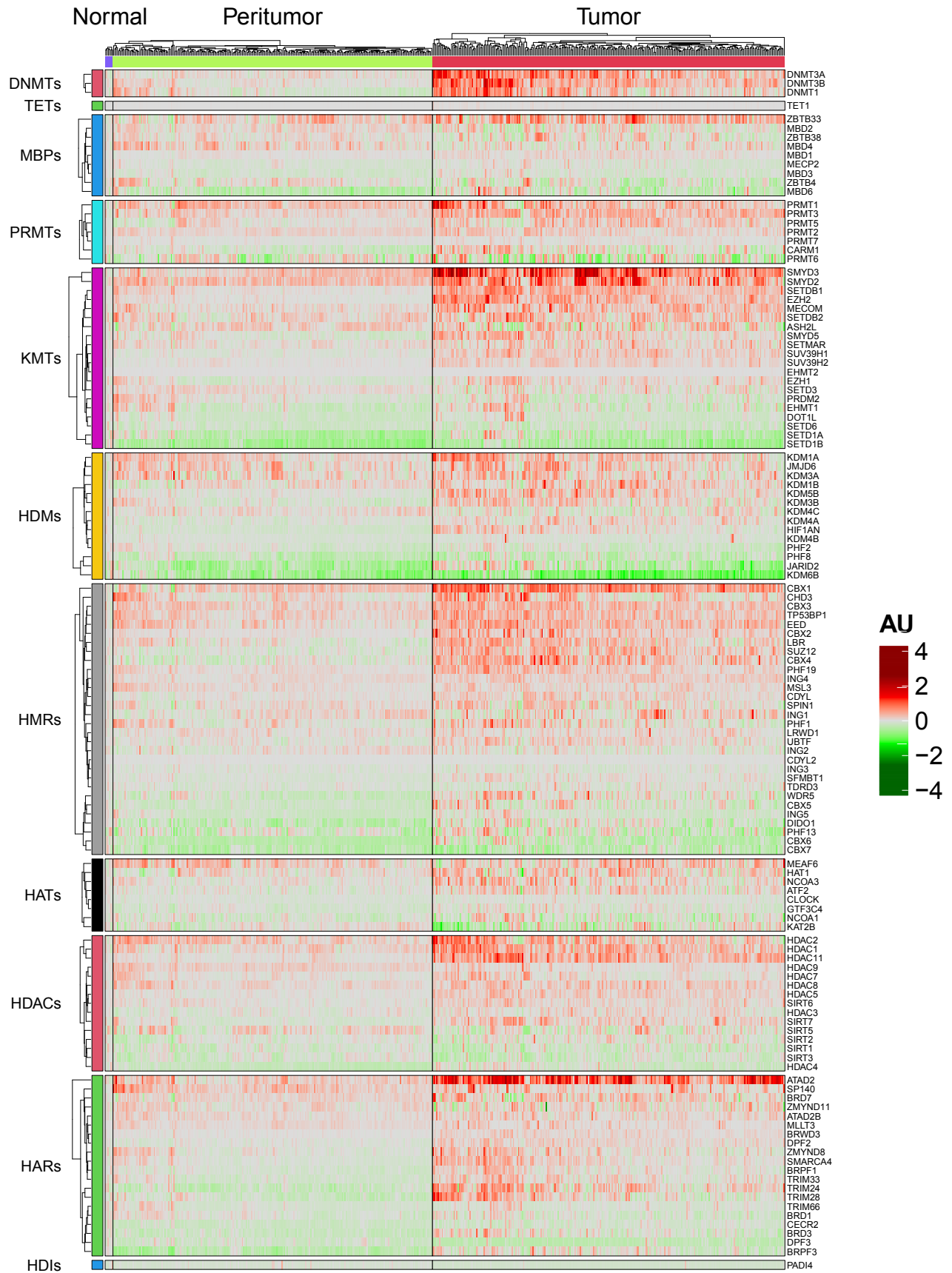

Supplement: Supplementary file 1 — (PDF 14.1 mb) [file 13105_2023_976_MOESM1_ESM.zip › 13105_2023_976_MOESM1_ESM/Suppl. Fig. 7A_ESM.pdf]

**B**

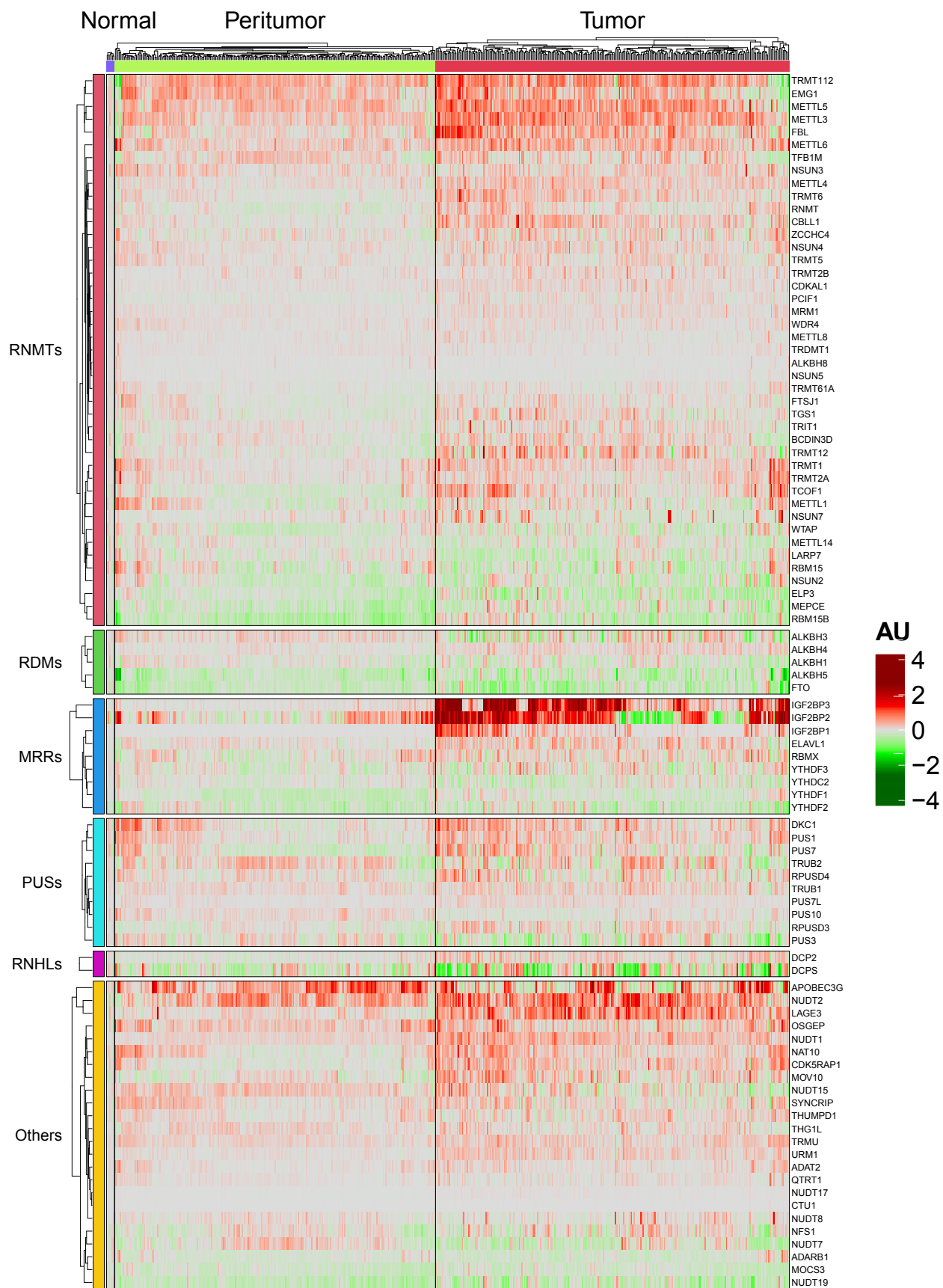

Supplement: Supplementary file 1 — (PDF 14.1 mb) [file 13105_2023_976_MOESM1_ESM.zip › 13105_2023_976_MOESM1_ESM/Suppl. Fig. 7B_ESM.pdf]

C

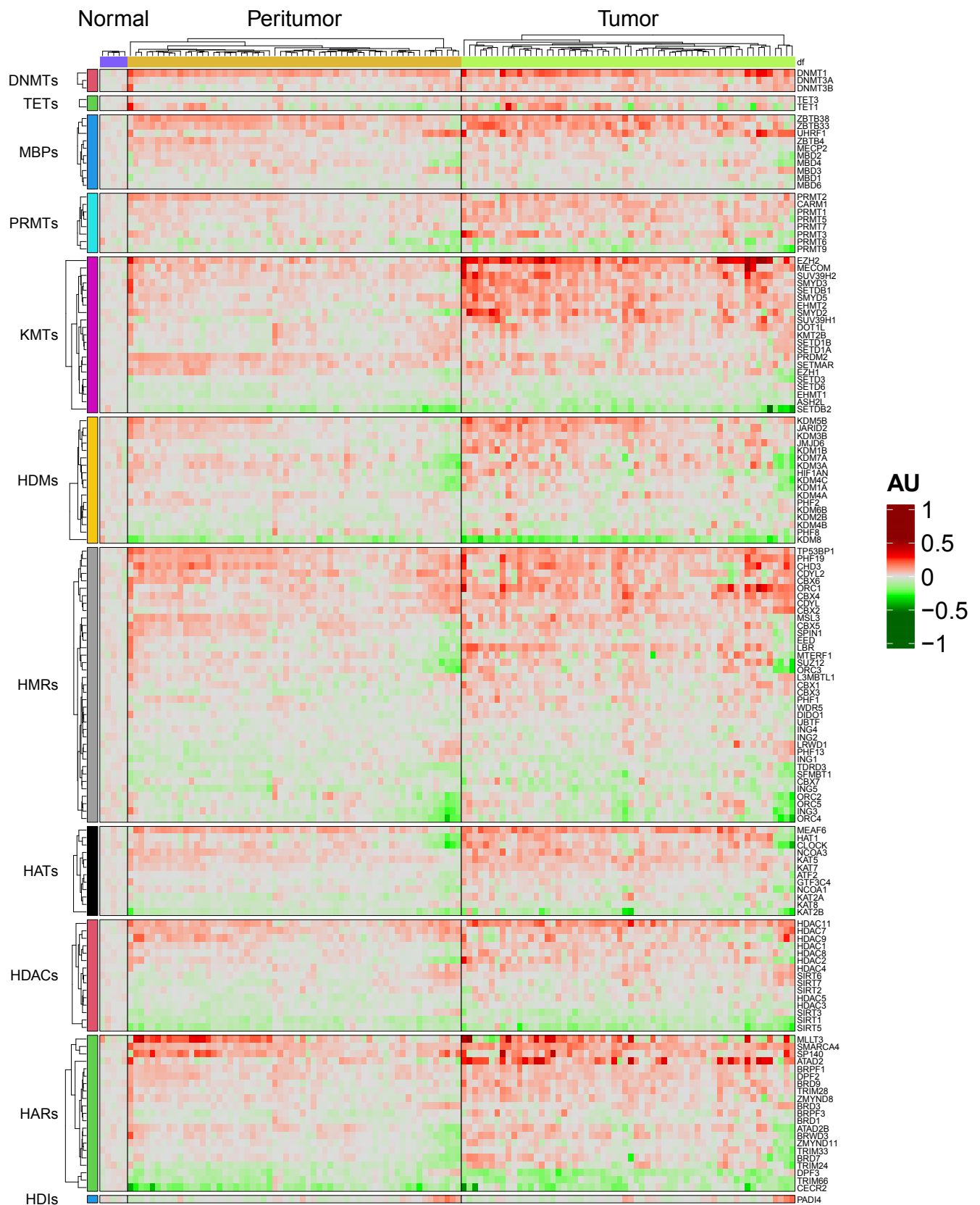

Supplement: Supplementary file 1 — (PDF 14.1 mb) [file 13105_2023_976_MOESM1_ESM.zip › 13105_2023_976_MOESM1_ESM/Suppl. Fig. 7C_ESM.pdf]

D

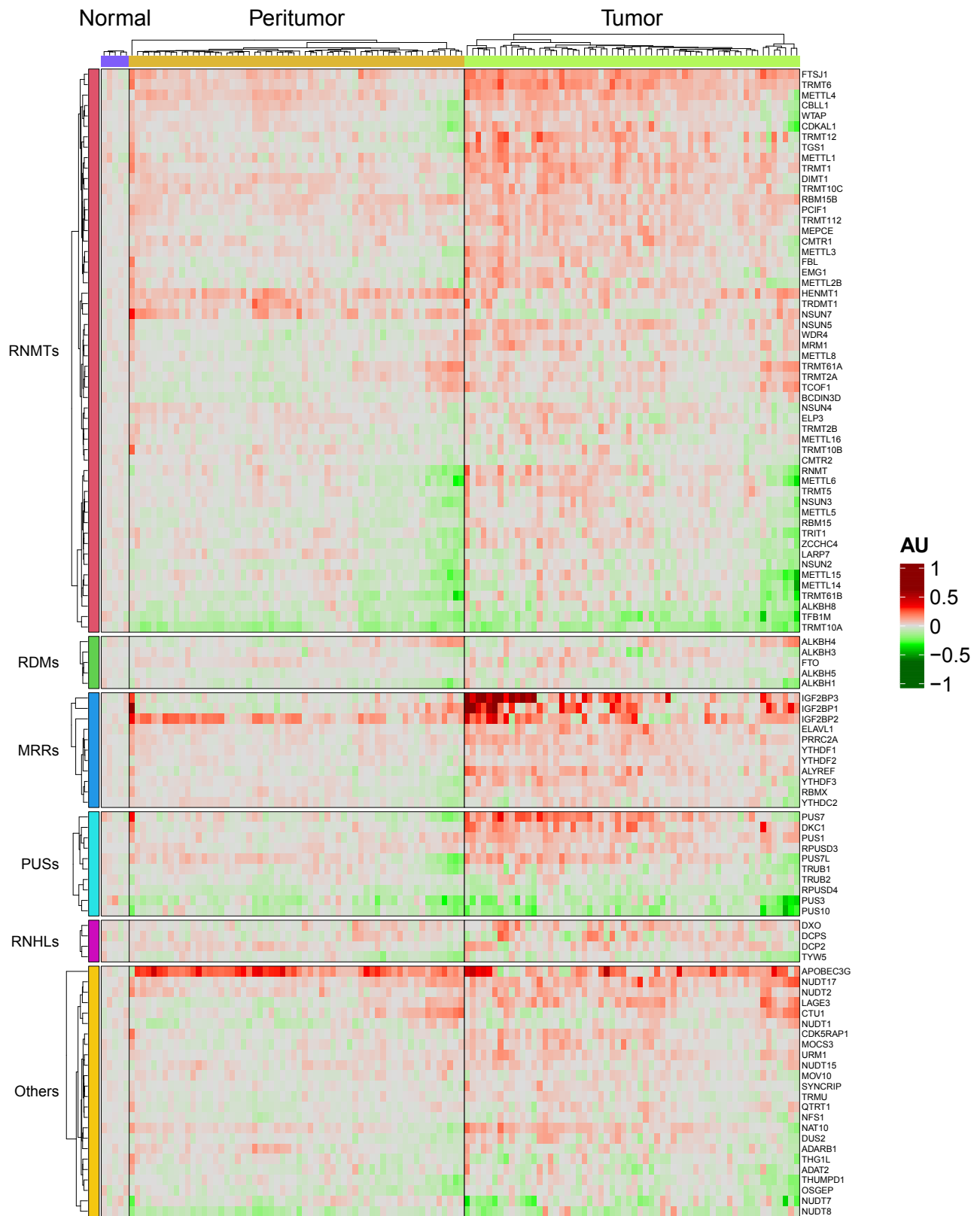

Supplement: Supplementary file 1 — (PDF 14.1 mb) [file 13105_2023_976_MOESM1_ESM.zip › 13105_2023_976_MOESM1_ESM/Suppl. Fig. 7D_ESM.pdf]

**A**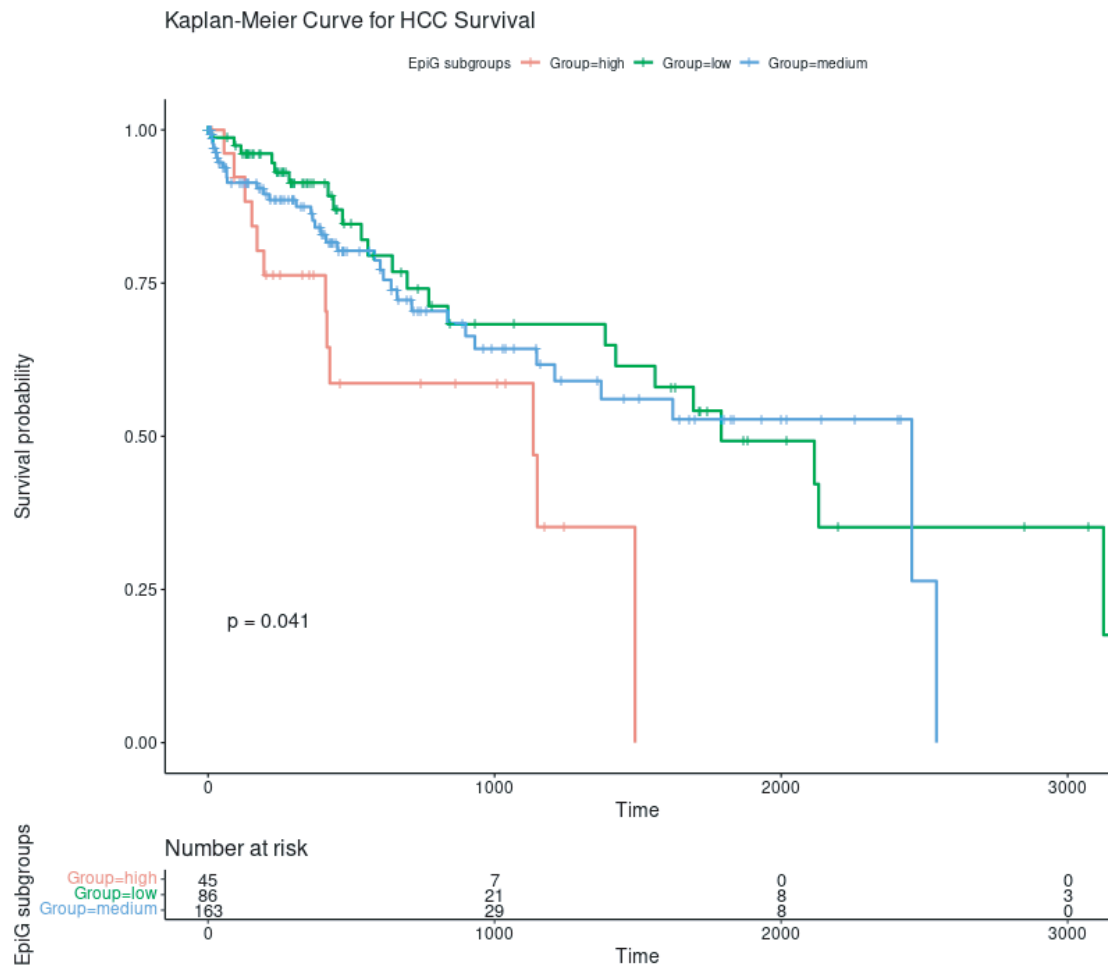**B**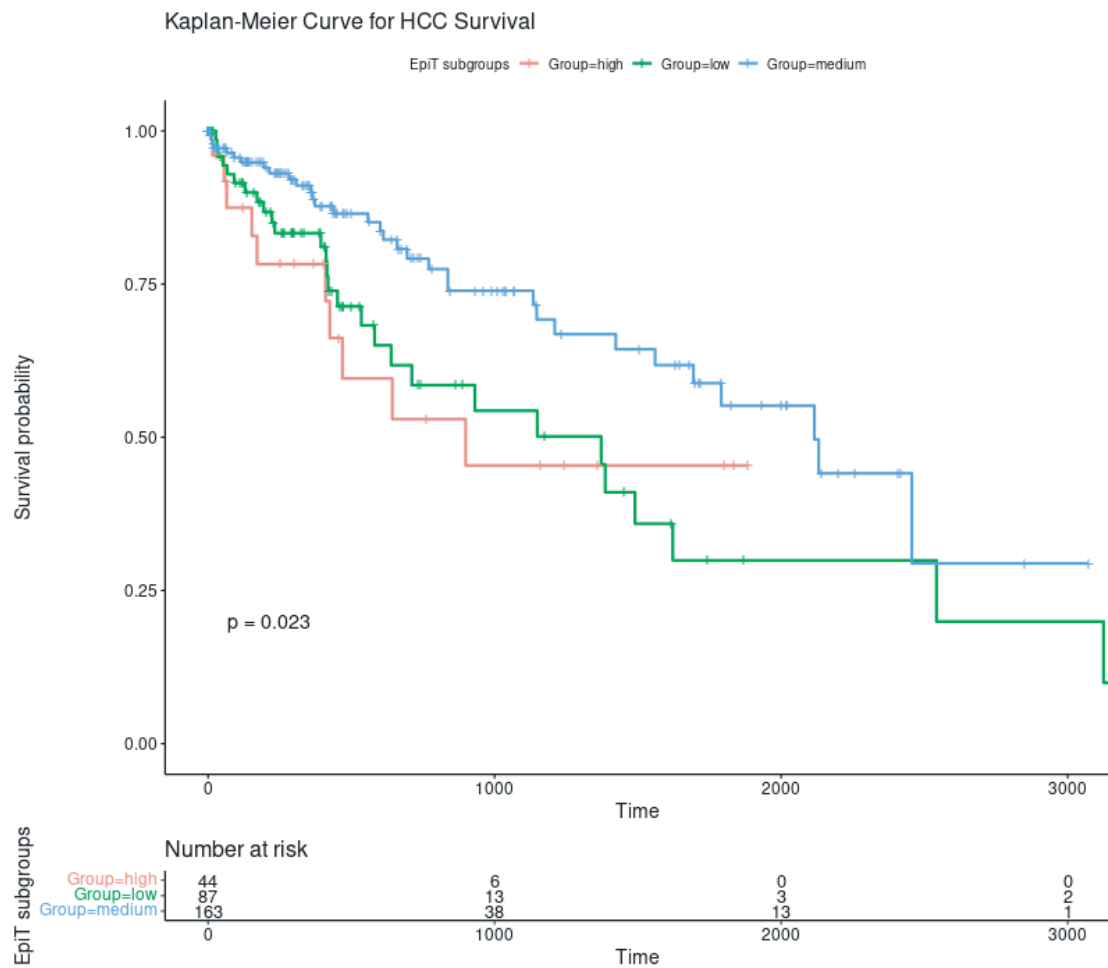

Supplement: Supplementary file 1 — (PDF 14.1 mb) [file 13105_2023_976_MOESM1_ESM.zip › 13105_2023_976_MOESM1_ESM/Suppl. Fig. 8_ESM.pdf]
